# Genome-wide identification and expression analysis of bHLH gene family revealed their potential roles in abiotic stress response, anthocyanin biosynthesis and trichome formation in *Glycyrrhiza uralensis*

**DOI:** 10.3389/fpls.2024.1485757

**Published:** 2025-01-21

**Authors:** Guohua Ding, Yanping Shi, Kerui Xie, Hongbin Li, Guanghui Xiao

**Affiliations:** ^1^ College of Life Sciences, Shaanxi Normal University, Xi’an, China; ^2^ College of Life Sciences, Key Laboratory of Xinjiang Phytomedicine Resource and Utilization of Ministry of Education, Shihezi University, Shihezi, China

**Keywords:** *bHLH* gene family, licorice, evolutionary analyses, expression patterns, stress responses, phytohormones

## Abstract

**Introduction:**

Licorice stands out as an exceptional medicinal resource with a long history of application, attributed to its substantial pharmacological potential. The basic helix-loop-helix (bHLH) transcription factors (TFs) gene family, being the second-largest in plants, is vital for plant development and adapting to environmental shifts. Despite this, the comprehensive characteristics of licorice *bHLH* gene family are not well-documented.

**Results:**

In this study, a detailed and thorough genome-wide identification and expression analysis of *Glycyrrhiza uralensis bHLH* gene family was carried out, resulting in the identification of 139 licorice *bHLH* members. Our duplication analysis highlighted the significant contribution of segmental duplications to the expansion of *G. uralensis bHLH* genes, with *GubHLH* genes experiencing negative selection throughout evolution. It was discovered that GubHLH64 and GubHLH38 could be importantly linked to the licorice trichome initiation and anthocyanin biosynthesis and GubHLH64 was also involved in the abiotic stress response. Additionally, certain subfamily III (d+e) GubHLH members could be implicated in the licorice drought response. GubHLH108, GubHLH109, and GubHLH116 were suggested to form a tightly related cluster, initiating transcriptional responses via JA signaling pathway.

**Discussion:**

In summary, our findings furnish a foundational understanding for future investigations of *GubHLH* gene functions and regulation mechanisms, shedding light on the potential applications of licorice in medicine and agriculture.

## 1 Introduction

*Glycyrrhiza uralensis* Fisch. (*G. uralensis*), which belongs to the Leguminosae family, is a perennial herb naturally thriving in sandy or saline soil within arid and semiarid regions of North China, Northeast China, Northwest China, and several other countries in Central Asia and Europe (Ding et al., 2022). With its history spanning more than 2000 years, *G. uralensis* has been a key component in traditional Chinese medicine. Initially documented in “Shen Nong’s Materia Medica”, this herb has found applications in 29.87% of preparations listed in the Pharmacopoeia of the People’s Republic of China (2020 edition), showcasing its significance (Jiang et al., 2020; Ding et al., 2022). It has been demonstrated to possess capabilities in invigoration, detoxification, analgesia, relieving coughs and harmonizing the effects of other medicines in traditional medicine (Li T. et al., 2020; Yang et al., 2015). Modern pharmacological studies have found that various active components, mainly flavonoids and triterpenoids, contribute to diverse pharmacological benefits in licorice (Hosseinzadeh and Nassiri-Asl, 2015). Licorice compounds have also shown promise as inhibitors of COVID-19 (Kim et al., 2023). Beyond medicinal applications, licorice and its extracts have found use in industries like food, tobacco, and household chemicals (Kao et al., 2014).

Acting as the central switches of all cellular regulation networks, TFs can bind the proper DNA elements and recruit additional proteins to perform transcription transactions, in turn, modulating gene expression and numerous biological processes involving plant growth and environment adaptation (Strader et al., 2022). Among TFs, the bHLH family comprises the second-largest plant gene family following the MYB gene family, playing pivotal roles (Feller et al., 2011). The first bHLH motif, with the capacity of dimerization and DNA binding, was identified in murine E12 and E47 proteins (Murre et al., 1989). The plant bHLH protein was originally found to be engaged in the maize anthocyanin synthesis (Ludwig et al., 1989).

The bHLH TFs derive their name from the structural characteristics of their conserved bHLH domain, approximately 50-60 amino acid residues in length, which is composed of two linked subregions. The N-terminal basic region with the length of 15-20 amino acids serves as a DNA-binding domain being in charge of recognization and binding of TFs to appropriate DNA sequences (Atchley et al., 1999). And they have been shown to recognize the hexanucleotide *cis*-regulatory element called E-box (5′-CANNTG-3′) in target gene promoter regions through certain conserved residues within the basic region, with G-box (5’-CACGTG-3′) being the commonest form (Li et al., 2006). The other one is the C-terminal HLH region with variable length of 40-50 amino acids, which form two α-helices with a variable length intervening loop by hydrophobic residues, facilitating the homodimers or heterodimers formation as well as the regulation of target genes (Murre et al., 1989; Nair and Burley, 2000). Whereas sequences of bHLH TFs outside the conserved domain represent considerable diversity (Atchley et al., 1999). The bHLH TFs in metazoans were reported to be classified into 6 main groups (A-F) according to their DNA contact motifs, evolutionary relationships, the preferent DNA binding sequence and function properties (Simionato et al., 2007). In plants, the majority (more than 50%) of bHLHs evolved from group B, and a few of them (8%) own the group A characteristics, while group C, E, and F characteristics are absent in plant bHLHs. There are also some plant proteins (11%) absent in animals (Pires and Dolan, 2010). Owing to the large variation, the plants bHLHs cannot be classified clearly complying with the classification in animals. In current studies, plant bHLHs are usually classified based on the results of Pires and Dolan (2010) and Carretero-Paulet et al. (2010), in which bHLHs were clustered into 26 and 32 subfamilies, respectively.

Plant bHLH TFs have been shown to be responsible for heterogeneous growth and metabolic processes, including seed germination (Oh et al., 2006), flowering (Kumar et al., 2012), stomatal fate (Raissig et al., 2016) and the development of awn and grain (Luo et al., 2013). It has also been widely reported that bHLH TFs are associated with the trichome development. The bHLH TF CsGL3 in tea is involved in the trichome regulation through forming complex with MYB TF CsMYB1 and CsWD40 (Li et al., 2022). A bHLH TF in *Artemisia annua*, AaMYC2-type, was found to play an important part in regulating trichome development (Khan et al., 2024). Tomato SlMYC1 plays a vital role in type VI glandular trichomes formation (Xu et al., 2018). Meanwhile, previous studies have highlighted the engagement of bHLH TFs in secondary metabolites biosynthesis such as artemisinin (Yuan et al., 2023) and triterpene saponin (Mertens et al., 2016). In *G. uralensis*, the GubHLH3 was reported to play a role in soyasaponin biosynthesis, which is a kind of triterpenoids (Tamura et al., 2018). Furthermore, since the first plant bHLH protein was identified to regulate anthocyanin synthesis in maize, a growing comprehension of bHLH participating in anthocyanin production regulation has been reported. The apple MdbHLH3 was validated to bind to anthocyanin biosynthesis related genes promoters, leading to enhanced anthocyanin content and fruit coloration (Xie et al., 2012). The mulberry bHLH3 is an essential regulator in anthocyanin biosynthesis and flavonoid homeostasis, thus affecting the fruit coloration (Li H. et al., 2020). The bHLH TF VvMYC1 was found to take part in the transcriptional cascade regulating the anthocyanin accumulation in *Vitis vinifera* (Hichri et al., 2010). The bHLH proteins TT8 (Transparent Testa8, AtbHLH42), GL3 (Glabra3, AtbHLH1) and EGL3 (Enhancer of Glabra3, AtbHLH2) are critical for trichome formation and anthocyanin biosynthesis in *Arabidopsis thaliana* (Qi et al., 2011). And the AtGL3 homologue in *Brassica napus*, BnGL3-1 owns the conserved functions as AtGL3, promoting anthocyanin production and flowers trichome formation (Gao et al., 2018). The bHLH2 in *Ipomoea purpurea* modulates seed trichome formation, proanthocyanidin and phytomelanins for pigmentation in seeds, as well as anthocyanin production (Park et al., 2007). FhGL3L and FhTT8L, two IIIf subfamily bHLHs from *Freesia hybrida*, were confirmed to partake in regulating the trichome patterning and anthocyanin accumulation (Li Y. et al., 2016). Moreover, the bHLHs have been found to exert functions in the regulation of environmental and abiotic responses. Overexpression of *AtbHLH68* can enhance adaptation to drought via ABA signaling in *Arabidopsis* (Le Hir et al., 2017). TabHLH49 in wheat can improve drought tolerance via regulating the dehydrin gene (Liu et al., 2020). TabHLH27-A1 was identified to balance drought tolerance and growth, enhancing wheat water use efficiency (Wang et al., 2024). Overexpressed *ZmPTF1* in maize improve the drought resistance, along with the enhanced yield and abscisic acid (ABA) synthesis (Li et al., 2019). OsbHLH062 in rice controls the transcription of target ion transporters in response to JA treatment, thereby altering the salt tolerance (Wu et al., 2015). The CabHLH035 in *Capsicum annuum* can increase salt tolerance by lowering intracellular Na^+^/K^+^ ratio and enhancing proline biosynthesis (Zhang et al., 2022). AhbHLH121 can improve salt tolerance by improving the antioxidant enzyme activity in peanut (Zhao et al., 2024). FIT (bHLH29, FER-like Iron Deficiency Induced Transcription Factor), a bHLH member from IIIa subfamily, interacts with bHLH38, bHLH39, bHLH100 and bHLH101, playing a core role in Fe deficiency response and homeostasis (Wang et al., 2013).

In the wake of the accomplishment of whole-genome sequencing and assembly for various plant species, bHLH proteins in multitudinous plants have been characterized. There were 162 *bHLH* members in *Arabidopsis* (Bailey et al., 2003), 251 in *Brassica rapa* (Miao et al., 2020), 440 in *Brassica napus*, 268 in *Brassica oleracea*, 208 in maize (Zhang et al., 2018), 167 in rice (Li et al., 2006), 437 in cotton (Lu et al., 2018) and 319 in soybean identified (Hudson and Hudson, 2015). A comprehensive genome-wide identification of *G. uralensis bHLH* gene family has not been explored. This study systematically analyzed the *G. uralensis bHLH* TF gene family, identifying 139 GubHLH members. And their physicochemical properties, evolution relationship and expression patterns were further investigated, providing a meaningful understanding for the characteristics and functions of the *G. uralensis bHLH* gene family.

## 2 Material and method

### 2.1 Identification of *G. uralensis bHLH* genes and analysis of their sequences

The whole-genome sequence of *G. uralensis* was assembled by Wuhan Benagen Technology Co., Ltd. (Wuhan, China). The bHLH domain (PF00010) hidden Markov model (HMM) was downloaded from the Pfam database (http://pfam.xfam.org/, accessed on 18 April 2023) and employed to search against the local *G. uralensis* protein database through HMMER v3.2 (http://hmmer.janelia.org/) software with the e-value of 10^-5^. Furthermore, the protein sequences of *Arabidopsis* bHLHs members were acquired from the *Arabidopsis* resource database (https://www.arabidopsis.org/, accessed on 18 April 2023) and used as the queries for a local BLASTP (blast v2.9.0) search against the *G. uralensis* protein database. Then the hits from two ways of search methods were combined and redundant genes were eliminated to create the GubHLH candidates. Then the NCBI Conserved Domain Database (CDD, https://www.ncbi.nlm.nih.gov/cdd/, accessed on 19 April 2023) and the SMART (http://smart.embl-heidelberg.de/, accessed on 19 April 2023) were utilized to verify the candidates with the bHLH domain. Subcellular localization predictions were performed using Plant-mPLoc program in Cell-PLoc 2.0 (Chou and Shen, 2008), and their physicochemical parameters were predicted using the ExPASy ProtParam tool (Gasteiger et al., 2003).

### 2.2 Phylogenetic analysis and classification of GubHLHs

The protein sequences of *G. uralensis* bHLHs and *Arabidopsis* bHLHs were combined and a multiple sequence alignment was generated using ClustalX v1.83 software (Thompson et al., 1997). A phylogenetic tree on the basis of the alignment was constructed by the MEGA7.0 employing the Neighbor-Joining (NJ) method. The parameter bootstrap was set as 1000 and amino acid substitution model was Jones-Taylor-Thornton (JTT) model (Kumar et al., 2016). The phylogenetic tree was then edited and visualized by iTol v5 (https://itol.embl.de/) (Letunic and Bork, 2021). The conserved bHLH domains sequences of GubHLH proteins were aligned and edited through Jalview v2.11.2.0 software (Waterhouse et al., 2009). The GubHLH domains logos were generated using TBtools v1.09876 (Chen et al., 2020). Tertiary structures of GubHLH bHLH domain were predicted through homology modeling on the SWISS-MODEL website (https://www.swissmodel.expasy.org/) website and visualized by PyMOL v2.4.0 (Seeliger and de Groot, 2010).

### 2.3 Gene structure, conserved motif and conserved domains analysis of the GubHLHs

Information regarding the intron-exon distribution of *G. uralensis* bHLH genes was extracted from the *G. uralensis* GFF annotation file. The conservative motifs in *GubHLH* proteins were identified utilizing the MEME online program v5.5.2 (Bailey et al., 2015). The motif number was 10 with the width from 6 to 50 residues. The analysis of conserved domains in *G. uralensis* bHLH proteins was conducted via the CDD. These results were visualized by TBtools v1.09876 (Chen et al., 2020). The GubHLH proteins secondary structures were analyzed through SOPMA server (Geourjon and Deléage, 1995).

### 2.4 Chromosomal distribution and gene duplication analysis

The physical location information of *GubHLH* genes on *G. uralensis* chromosomes was retrieved from the GFF file and visualized by TBtools. Duplication events analysis of *GubHLH*s was performed based on their amino acid sequences and chromosome locations utilizing Multicollinearity Scanning Toolkit (MCscanX) software (Wang et al., 2012), with visualizations and editing accomplished using Advance Circos program within TBtools v1.09876. *Arabidopsis* genomic data were accessed from the TAIR10 website (http://www.arabidopsis.org/index.jsp). Rice, maize, *M. truncatula* and *Glycine max* genomic data were downloaded from the JGI website (https://phytozome-next.jgi.doe.gov/). Collinearity analysis of licorice with these five species was performed by the One Step MCScanx program and visualized through TBtools Dual Systeny Plotter program in TBtools v1.09876. The synonymous substitution rate (Ks), nonsynonymous substitution rate (Ka), and Ka/Ks ratio between homologous *GubHLH* gene pairs were calculated applying KaKs_Calculator v3.0 through the NG method (Zhang, 2022).

### 2.5 *Cis*-regulatory elements analysis in *GubHLHs* promoters

The 2000 bp upstream sequences of *GubHLH* coding sequences were retrieved using TBtools, relying on the *GubHLHs* DNA sequences, which were then submitted to the PlantCare database for the prediction of *cis*-elements in the *GubHLHs* promoters (accessed on 17 June 2023) (Lescot et al., 2002).

### 2.6 Functional enrichment and PPI analysis

The protein sequences of GubHLHs were submitted to KOBAS3.0 (accessed on 20 June 2023) (Bu et al., 2021) and the KEGG database (https://www.kegg.jp/, accessed on 20 June 2023) to acquire the Gene Ontology (GO) terms and KEGG pathway annotations, respectively. Additionally, protein-protein interaction (PPI) analysis was conducted on the STRING website using the orthologs in *Arabidopsis* as references (http://string-db.org, accessed on 26 September 2023) (Szklarczyk et al., 2023).

### 2.7 Subcellular localization verification

The coding sequence of *GubHLHs* without termination codons were cloned into pCambia1300-GFP vector to construct the recombinant vectors, which was then transformed into *Agrobacterium* strain GV3101 and infiltrated into *Nicotiana benthamiana* leaves along with the nuclear marker (GhBES1-mCherry) strain. The empty pCambia1300-GFP vector was served as the control. The fluorescence information was obtained through laser confocal microscopy (Leica TCSSP8, Wetzlar, Germany).

### 2.8 Yeast two hybrid assays

The *GuMYB75* and *GuTTG1* were obtained as the orthologs of *AtMYB75* and *AtTTG1* through the BLASTP search against the *G. uralensis* genome database using *AtMYB75* and *AtTTG1* as queries, respectively. And the genes with the highest bitscores were chosen. The full-length CDSs of *GubHLH64*, *GubHLH38* and *GuMYB75* were individually incorporated into the pGADT7 vectors as bait construct. Meanwhile, *GuMYB75* and *GuTTG1* were cloned into pGBKT7 vectors as prey construct. The bait and prey vectors were then co-transformed into the Y2HGold yeast strain and selected on synthetic dropout (SD) medium lacking leucine (-L) and tryptophan (-T). The transformed yeast cells on the SD-T-L mediums were isolated and dotted on SD-T-L-H (histidine) and SD-T-L-H-A (adenine) medium. The primers were listed in **Supplementary Table S1**.

### 2.9 Plant materials and treatments

The seeds of licorice underwent dormancy breakage through a 50-minute treatment with 98% concentrated H_2_SO_4_, followed by three washes with sterilized distilled water. Subsequently, the treated seeds were germinated and cultured in plastic pots filled with nutrient soil and vermiculite mixed in the proportion of 2:1 (v:v) within an automatic climate chamber maintaining steady conditions of 16 h/28°C at the day and 8 h/25°C during night as well as the relative humidity of 50-55%. After 60 days of cultivation, the seedlings were transferred to Hoagland solution medium containing 10% PEG6000 and 150 mM NaCl for abiotic treatment. Licorice roots were collected at different time points after NaCl and drought treatment (0 h, 2 h, 6 h, and 12 h). Simultaneously, 60-day-old seedlings were subjected to treatments with 50 mM ABA, 100 µM auxin (IAA), 100 µM gibberellin (GA), and 100 µM methyl jasmonate (MeJA) in the Hoagland solution medium for varying durations (0 h, 2 h, 6 h, and 12 h). Three biological replicates were collected for each experimental condition, with each replicate comprising roots from 15 seedlings, which were then promptly frozen in liquid nitrogen and stored at -80°C.

### 2.10 Quantitative Real-time PCR (qRT-PCR) analysis

The RNA-seq raw data of *G. uralensis* roots under drought treatment were retrieved from the National Center for Biotechnology Information (NCBI) database (BioProject number: PRJNA810509) (Yao et al., 2022) and utilized to assess the expression levels of *G. uralensis bHLH* genes. After trimming and quality control by fastp v0.19.5 (Chen et al., 2018), the clean data were mapped to the *G. uralensis* genome using the HISAT2 software v2.1.0 (Kim et al., 2019). And RSEM software v1.3.3 (Li and Dewey, 2011) was used to obtain the gene read counts and normalize the expression to transcripts per million (TPM) values. DESeq2 (v1.10.0) (Love et al., 2014) was used to perform the differential expression analysis with the criteria |log_2_ (Fold change)| > 1.0 and p values < 0.05. Total RNA from *G. uralensis* roots were extracted using the RNAprep Pure Plant kit (TIANGEN BIOTECH, Beijing, China) which were applied to synthesize the first strand cDNAs utilizing the EasyScript One-step gDNA Removal and cDNA Synthesis SuperMix (Vazyme, Nanjing, China) following the manufacturer’s instructions. Primer Premier 5.0 was applied to design the qRT-PCR primers which were listed in **Supplementary Table S** **2**. The qRT-PCR analysis were conducted on a Bio-RAD CFX96 Real-Time system (Hercules, CA, USA) using the 2×ChamQ SYBR qPCR Master Mix (Vazyme, Nanjing, China). The expression levels of *GubHLHs* were calculated using 2^−ΔΔCt^ method with *Guactin* (NCBI accession number: EU190972.1) as an internal control.

## 3 Results

### 3.1 Identification of licorice bHLH members and their physicochemical properties

Through searches against the *G. uralensis* whole-genome protein database based on the hidden Markov model called bHLH domain (PF00010) and the queries AtbHLH protein sequences as well as the subsequent bHLH domain integrality verification for the combined candidate GubHLH members, we obtained 139 *bHLH* gene family members in *G. uralensis* genome (**Supplementary Table S3**). They were then designated in the light of their positional order on *G. uralensis* chromosomes as *GubHLH1*-*GubHLH139*, and their sequences were list in **Supplementary Table S4**. Furthermore, the physicochemical properties of GubHLH proteins were analyzed. These GubHLH proteins varied from 73 (GubHLH69) to 777 (GubHLH10) amino acids in length, with molecular weights ranging from 8.29 kDa (GubHLH69) to 85.16 kDa (GubHLH61) and an average of 41.19 kDa. The theoretical isoelectric points (pI) of GubHLH proteins spanned from 4.55 (GubHLH49) to 10.29 (GubHLH87). The negative grand average of hydropathicity (GRAVY) values, distributing from -1.068 to -0.012, highlighted their hydrophilicity and solubility features which may correlate with their underlying function as TFs. The instability index predictions suggested that nearly all GubHLH proteins were likely unstable *in vitro*, with an instability index higher than 40, excluding GubHLH131, GubHLH127, and GubHLH59. And the aliphatic index values of GubHLH proteins were between 49.33 (GubHLH42) and 103.91 (GubHLH119), indicating a substantial variation in thermal stability. Moreover, the subcellular localization predictions showed that 138 out of the 139 GubHLH proteins were located in the nucleus, with GubHLH56 being found in both Golgi apparatus and nucleus (**Supplementary Table S3**).

### 3.2 Conserved amino acid residues in the bHLH domain of GubHLH proteins

**Figure 1:**
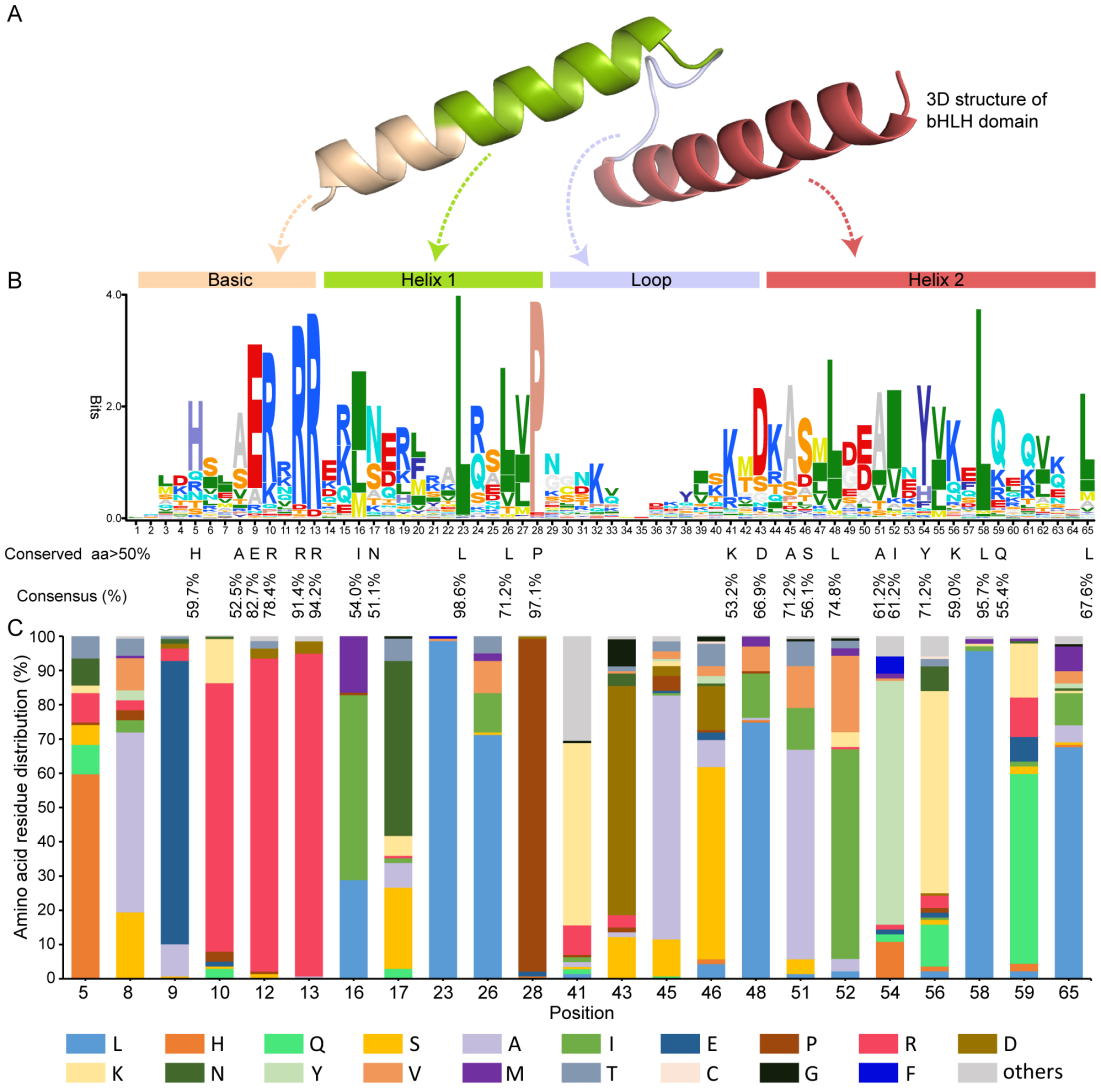
The bHLH domain sequences characterizations in the GubHLHs. **(A)** Three-dimensional structure of the GubHLH12 bHLH domain. **(B)** Sequence logo of conserved bHLH domain of GubHLHs. The amino acid height indicated the frequency of the sequence observed in GubHLH proteins at that site. The conservation sites with a consensus ratio exceeding 50% in the bHLH domain were highlighted by the black letters below. **(C)** The amino acids distribution at the 23 sites with a consensus ratio exceeding 50% in GubHLH bHLH domains. The numerals at the bottom represent the amino acid residues positions.

A multiple alignment analysis amino acid sequences in the bHLH domain of GubHLH proteins was performed to explore their features (**Supplementary Figure S1**). The analysis revealed that *G. uralensis* bHLH family proteins contained the typical conserved bHLH domain, and four conserved regions were presented within these domains, including one basic region, the first helix, the loop and the second helix region (**Figures 1A, B**). Within these bHLH domains, 23 conserved amino acids with the consensus ratio exceeding 50% existed and 7 out of them (Glu-9, Arg-10, Arg-12, Arg-13, Leu-23, Pro-28, Leu-58) exhibited a consensus ratio surpassing 75% (**Figures 1B, C**). The basic region being crucial for DNA-binding activity to target genes featured six conserved residues (His-5, Ala-8, Glu-9, Arg-10, Arg-12, and Arg-13) in GubHLH proteins. The first helix region contained 5 conserved amino acid residues (Ile-16, Asn-17, Leu-23, Leu-26, and Pro-28). The loop region exhibited conservation at Lys-41 and Asp-43. Ten residues (Ala-45, Ser-46, Leu-48, Ala-51, Ile-52, Tyr-54, Lys-56, Leu-58, Gln-59, and Leu-65) were identified to be conserved in the second helix region. Notably, the residues Leu-23 and Pro-28 showed particular high conservation within the 139 *G. uralensis* bHLH proteins, emphasizing their notability in the formation of bHLH proteins dimers.

### 3.3 Classification and phylogenetic relationships of GubHLHs

**Figure 2:**
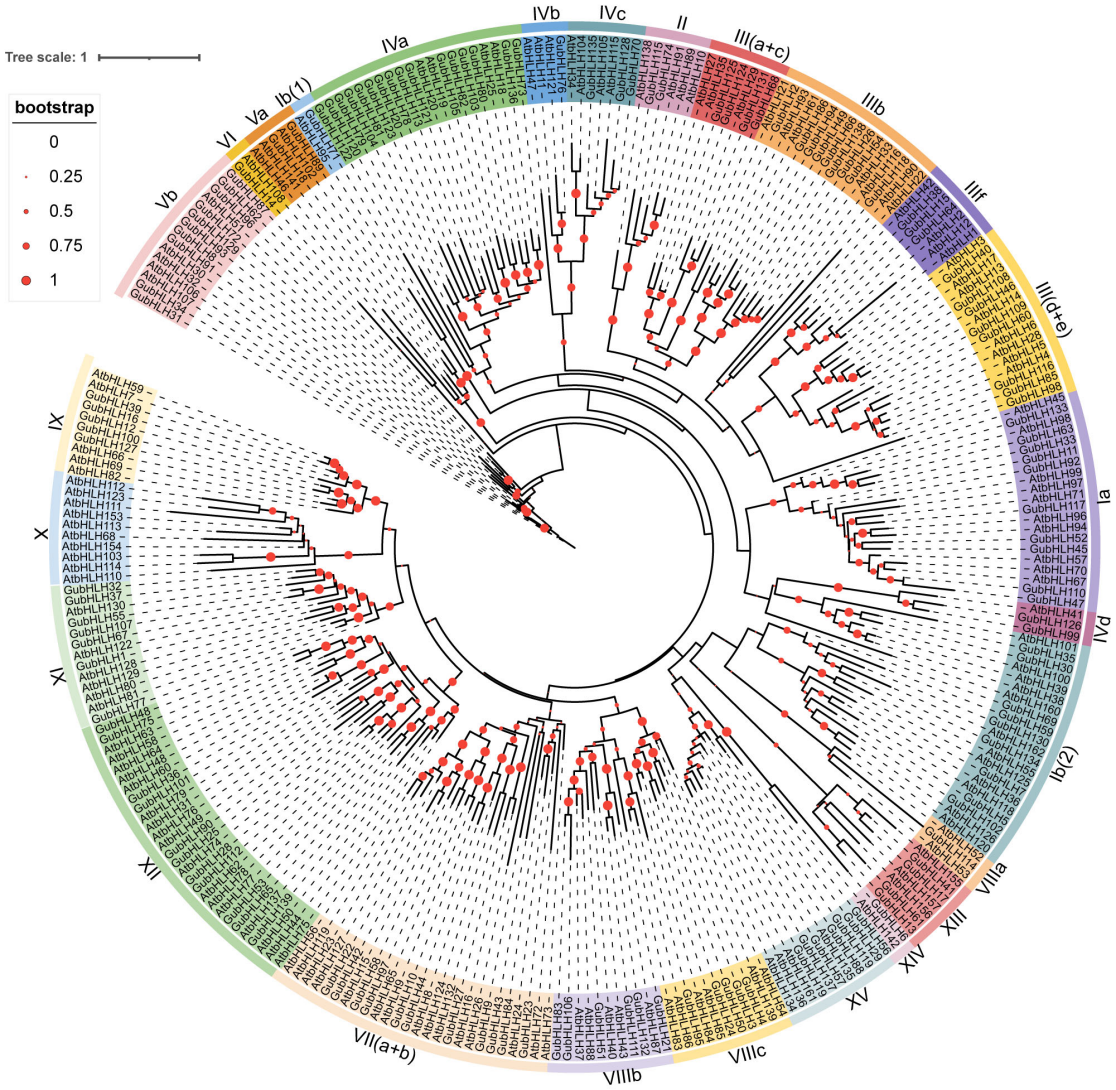
The phylogenetic tree of bHLH family members in *G. uralensis* and *A. thaliana*. Clustalx and MEGA 7 were used to generated the tree through NJ method with 1,000 bootstrap replicates. The bHLH proteins were clustered into different subgroups according to the priority classification rule of AtbHLHs, which was presented by differently colored branches.

To classify and examine the phylogenetic relationships among GubHLH members, a Neighbor-Joining (NJ) phylogenetic tree was constructed using the protein sequences of 139 bHLH proteins from *G. uralensis* and 144 from *A. thaliana*. As depicted in **Figure 2**, all bHLH proteins were organized into 26 subfamilies based on the tree’s topological structure and a classification method recommended by previous studies (Heim et al., 2003; Pires and Dolan, 2010), which include Ia, Ib (1), Ib (2), II, III (a+c), IIIb, III (d+e), IIIf, IVa, IVb, IVc, IVd, Va, Vb, VI, VII (a+b), VIIIa, VIIIb, VIIIc, IX, X, XI, XII, XIII, XIV and XV. And GubHLHs were distributed across 25 subfamilies, and notably, they did not cluster together with AtbHLHs in subfamily X. Among them, subfamily IVa contained the highest number with 16 GubHLHs, followed by subfamilies VII (a+b) and XII, each with 11 GubHLHs. While the subfamilies Ib (1), IVb, VI, VIIIa, and XIV contained only one GubHLH each, making them the smallest subfamilies (**Figure 2**; **Supplementary Figure S2**). Within most subfamilies, comparable numbers of bHLH proteins were distributed in *Arabidopsis* and *G. uralensis*. There are also varied abundance of bHLH protein between two species within some subfamilies, which might be attributed to the gene expansion or loss during evolution. Subfamily IVa and Vb contained four and five AtbHLH proteins, and the numbers of GubHLH proteins increased to sixteen and ten, respectively, indicating the possible expansion of the GubHLH proteins in IVa and Vb subfamily. The absence of subfamily X in *G. uralensis* might indicate the loss or undifferentiation of GubHLH proteins throughout evolution. And these results suggest that the bHLH members in IVa and Vb might have species-special functions in *G. uralensis* and the function of bHLHs in subfamily X might be replaced by other bHLHs.

### 3.4 Gene structure, conserved motif and domain analysis of GubHLHs

**Figure 3:**
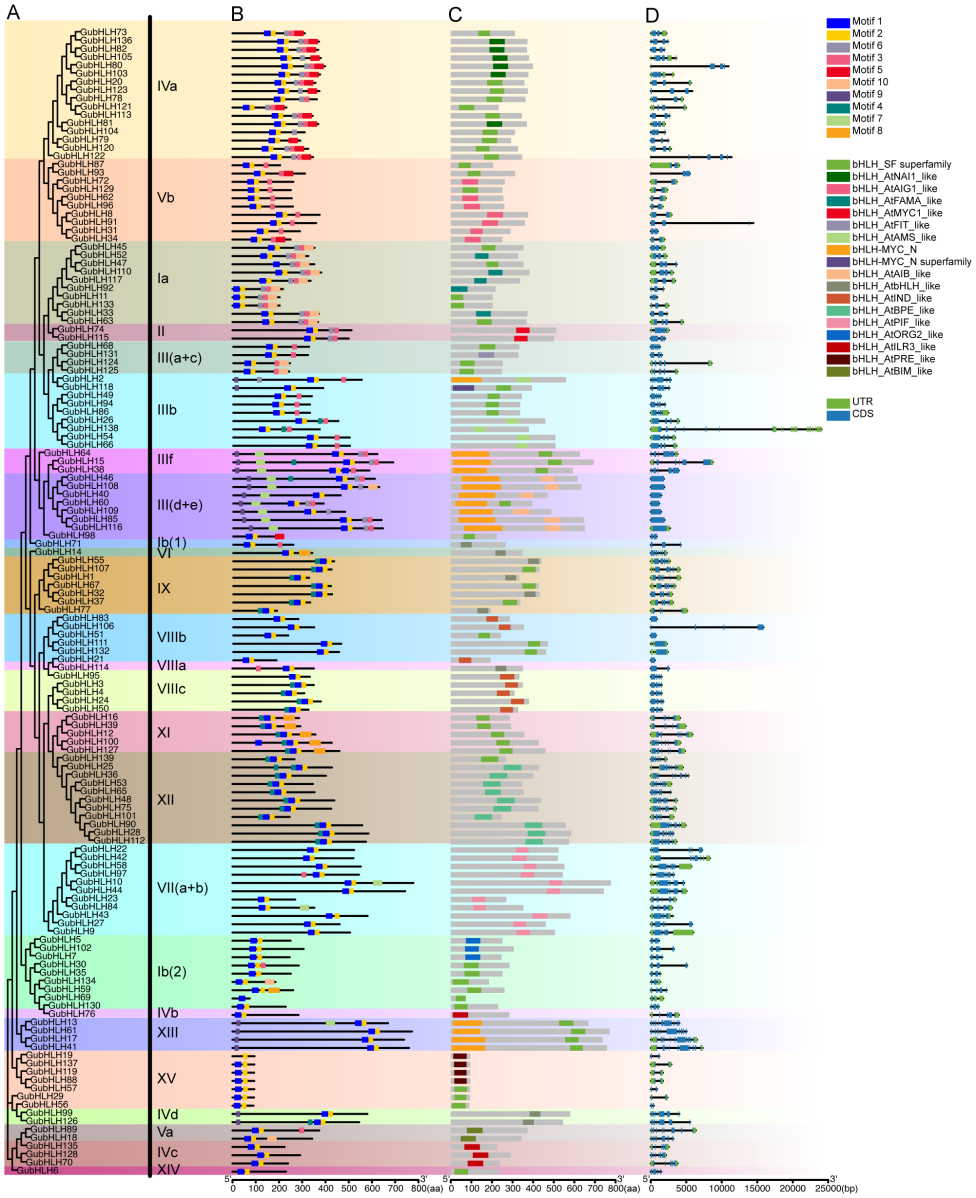
The phylogenetic tree, conserved motifs distribution, domains composition and gene structure of GubHLHs. **(A)** The neighbor-joining (NJ) phylogenetic tree of GubHLH proteins was generated using protein sequences with 1000 bootstrap replicates; **(B)** The distribution of conserved motifs in GubHLH proteins. **(C)** The distribution of bHLH domains in GubHLHs; **(D)** The gene structures of the GubHLHs, in which green rectangles indicated the untranslated regions (UTRs), blue rectangles indicated exons and black lines indicated the introns.

A phylogenetic tree was constructed using the 139 GubHLH protein sequences (**Figure 3A**), in which the classification of GubHLHs aligned with the results in **Figure 2**. To broaden the understanding of structural features of GubHLH proteins, the composition of conserved motifs, distribution of domains and exons/introns of GubHLHs were analyzed (**Figures 3B-D**). MEME results revealed 10 conserved motifs within the GubHLHs. The motifs sequences were submitted to the Pfam and InterProScan databases for annotation. Motifs 1 and 2 were annotated to the bHLH domain, and motifs 7 and 9 were bHLH-MYC and R2R3-MYB transcription factor N-termini (**Supplementary Table S5**). All GubHLH proteins contained bHLH domain related motifs. Motifs 7 and 9 were distributed in subfamilies III (d+e) and IIIf. GubHLH2, GubHLH118 and all GubHLHs in subfamilies XIII and IVd contained motif 9. The distribution of these motifs was generally conserved within subfamilies, indicating their potential functional similarities.

Meanwhile, the analysis of conserved domains using the CDD identified 18 types of bHLH domains in GubHLHs, highlighting their extensive diversity. Similar bHLH domain types were found within the same subfamily, corroborating the subfamily classification evidently in the phylogenetic tree (**Figure 3C**). GubHLH2, GubHLH118 and the GubHLH proteins in subfamilies III (d+e), IIIf and XIII were each characterized by the presence of a bHLH-MYC_N domain, strongly supporting the motif distribution in MEME results.

The exon-intron structures across *GubHLH* genes disclosed substantial variations. The subfamily XIII members had the highest exon count, featuring eleven exons in *GubHLH13/17/41* and ten in *GubHLH61*. Conversely, most members of subfamily III (d+e) and VIIIb each contained only one exon. The majority members in subfamily III (d+e) had no introns. Except for *GubHLH138* possessing 19 introns in subfamily IIIb, the subfamily XIII *GubHLH*s contained the greatest number of introns, ranging from 9 to 11. *GubHLH138* had the most number of UTRs, with one in 3’ UTR and 14 in 5’ UTR. The similar exons/introns distribution within subfamilies supported the notion that closely related evolutionary relationships are linked to similar gene structures.

Furthermore, the secondary protein structures of GubHLHs widely influencing protein sequence, structure, activity, stability, and abundance, were predicted and the results were exhibited in **Supplementary Table S6**. GubHLH proteins mainly consisted of alpha helix (16.44-67.39%), random coil (30.11-73.82%), extended strand (0-20.27%) and beta turn (0-6.46%). The VII (a+b) subfamily members owned the largest proportion of random coil (54.06-73.45%) and the minimum proportion of alpha helix (16.44-31.88%). While XV subfamily GubHLHs had the largest proportion of alpha helix (64.04-67.39%) and the minimum proportion of random coil (30.11-35.96%).

### 3.5 Chromosomal location and collinearity analysis of *GubHLHs*

The genomic distribution of *G. uralensis bHLH* genes was visualized on chromosomes, revealing an uneven distribution across 8 chromosomes (**Supplementary Figure S3**). Among them, chr2 owned the biggest amount of *GubHLHs* with 44 genes (31.65%). Chr1, chr6, chr5, chr7, chr3, chr4 and chr8 harbored 22, 22, 18, 17, 16, 16 and 3 *GubHLHs*, respectively.

**Figure 4:**
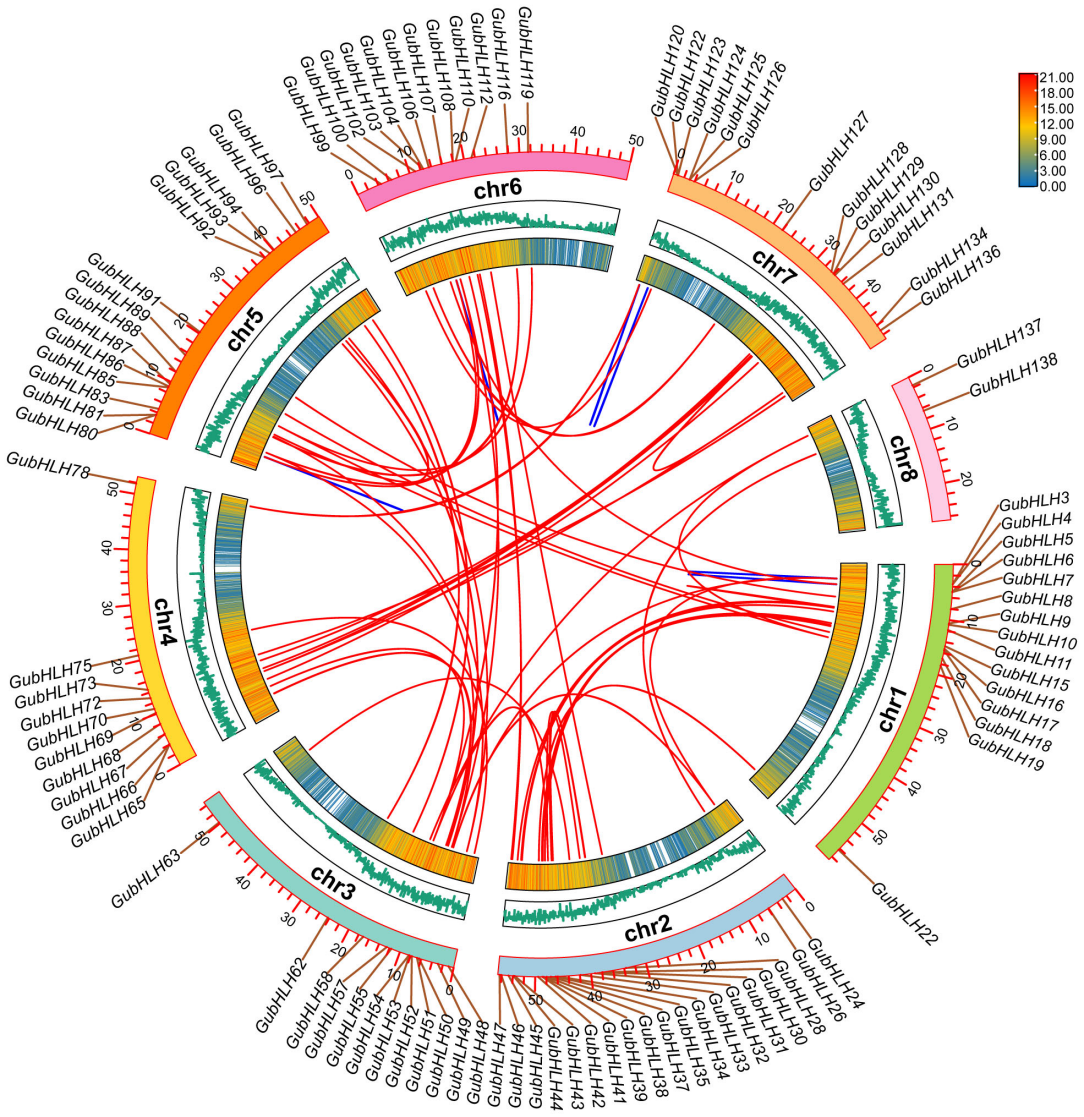
The collinearity of *GubHLHs* in *G. uralensis* genome. The blue lines represent the tandem duplication gene pairs and the red lines represent segmental duplication gene pairs relationship between *GubHLHs*.

Segmental and tandem duplications occupy important positions in gene family evolution. In the case of *GubHLHs*, 61 gene duplication events were identified, comprising 55 segmental and 6 tandem duplications. This suggested that segmental duplications were primarily responsible for the *GubHLH* gene family’ evolution (**Figure 4**; **Supplementary Table S7**). The calculation of the Ka/Ks ratio for these gene pairs yielded values less than 1, ranging from 0.045 to 0.566 (**Supplementary Table S8**). These findings implied the purifying selection throughout *GubHLH*s’ evolution, aiding in the preservation of their conserved structures.

For further exploration of the *GubHLH* gene*s* duplication timing and the underlying phylogenetic mechanism, collinearity analysis between *G. uralensis* and 5 representative species, including 2 monocots (rice and maize) and 3 dicots (*Arabidopsis*, *G. max* and *M. truncatula*) was performed (**Supplementary Figure S4**). The results revealed that a total of 127 *bHLH* genes of *G. uralensis* were collinearly associated with those of *G. max*, followed by 120 *GubHLH* genes with *M. truncatula*, 91 with *Arabidopsis*, 44 with *O. sativa*, and 34 with *Z. may* (**Supplementary Table S9**). Compared to other plants, *G. uralensis* shared significantly larger number of collinear pairs with *G. max* and *M. truncatula*, which belong to Fabaceae family along with *G. uralensis*. Additionally, 24 *GubHLHs* exhibited collinear associations with all other five species, which might have existed before their ancestral divergence and play important roles in bHLH evolution (**Supplementary Figure S5**).

### 3.6 Functional enrichment and PPI analysis

GO enrichment analysis was conducted to elucidate the potential functions of the GubHLHs, which were categorized into three main groups (**Supplementary Figure S6A**, **Supplementary Table S10**). In biological process, there were 109 GubHLHs enriched in regulation of biological process and biological regulation term, representing 78.42% of the GubHLHs. Metabolic process and cellular process were the second-largest terms, involving 107 genes each (76.98%). A total of 62 GubHLHs were enriched in single-organism process, 51 in response to stimulus, 48 in developmental process, 46 in multicellular organismal process, 27 in reproduction and reproductive process each, 18 in positive regulation of biological process, 17 in signaling, 12 in negative regulation of biological process and multi-organism process each, 7 in cellular component organization or biogenesis, 2 in growth and rhythmic process each. GubHLH106 was linked to localization. As for the molecular functions, almost all of the GubHLHs were enriched in binding except GubHLH14 and GubHLH22, followed by 133 GubHLHs in nucleic acid binding transcription factor activity and 2 GubHLHs in catalytic activity. In terms of cellular component, the majority GubHLHs were enriched in cell, organelle, and cell part, each with 138 members. And 138 out of 139 GubHLHs were enriched in nucleus, with the exception of GubHLH22. Seven GubHLHs were enriched in macromolecular complex and organelle part. GubHLH27 was enriched in cell junction and symplast.

Furthermore, KEGG enrichment analysis was performed (**Supplementary Figure S6B**). A total of 70 GubHLHs were enriched in the signal transduction pathway, 43 of which were engaged in MAPK signaling pathway and 64 of which were engaged in plant hormone signal transduction. Additionally, 42 GubHLHs were associated with environmental adaptation pathways, including 24 involved in plant-pathogen interaction and 18 involved in circadian rhythm (**Supplementary Table S11**).

**Figure 5:**
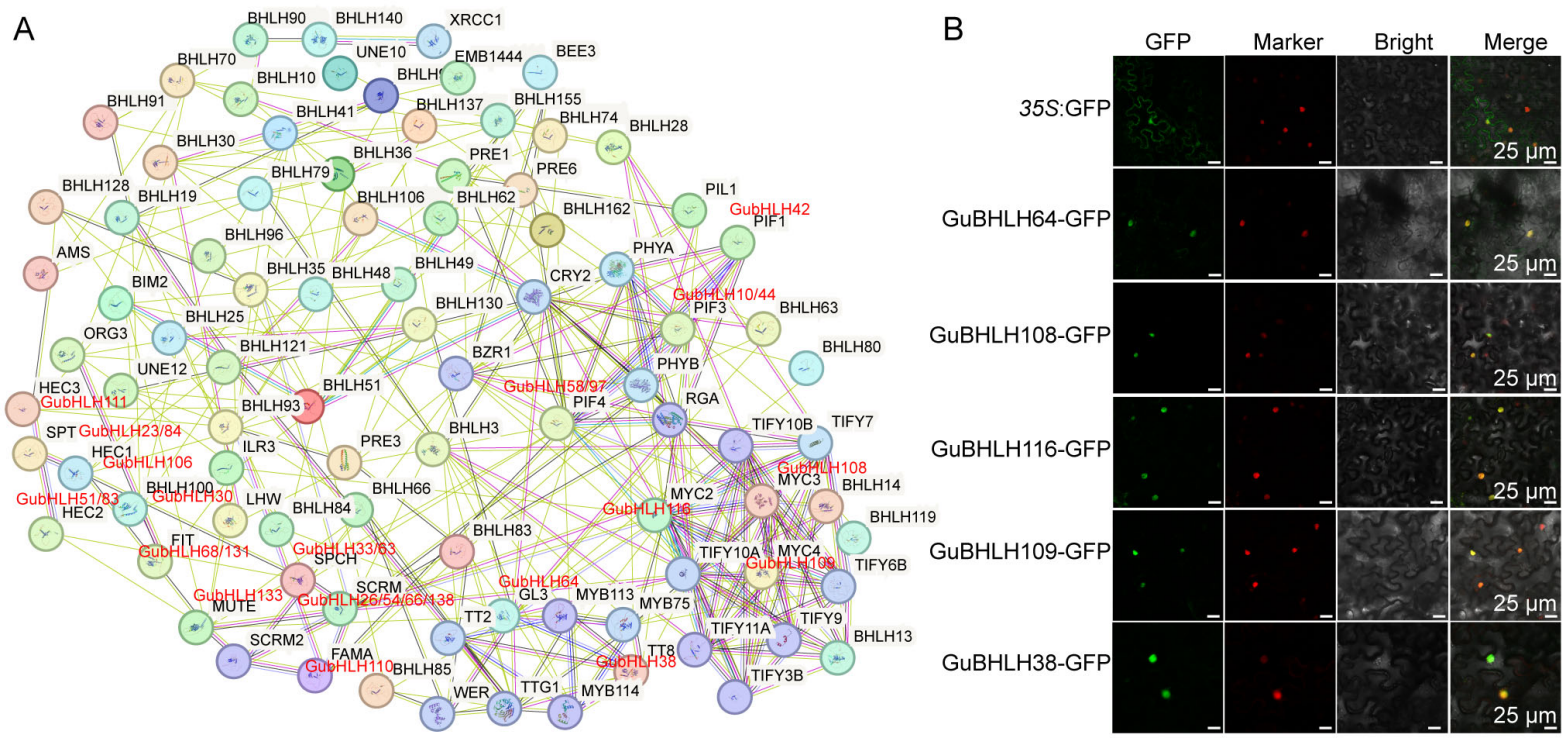
Protein-Protein interaction (PPI) network for GubHLHs and subcellular locations of GubHLH proteins. **(A)** PPI network for GubHLHs based on their orthologs in *Arabidopsis*. **(B)** Subcellular locations of GubHLH108, GubHLH109, GubHLH116, GubHLH64 and GubHLH38. The GhBES1-mCherry was used as the nuclear marker. The scale bars were 25 μm.

In addition, a PPI network was built to explore the potential interactions among the GubHLH proteins (**Figure 5A**). A total of 137 GubHLH proteins had homologous proteins in *Arabidopsis*, in which 89 GubHLHs were predicted to have interactions. Several noteworthy interactions were predicted. For instance, GubHLH58/97 (homologous with PIF4, Phytochrome Interacting Factor4) were predicted to interact with GubHLH42 (homolog of PIF1/PIL5, PIF3-Like 5, PIF1/bHLH015), GubHLH10/44 (homolog of PIF3), and phytochromes (PHYA and PHYB). GubHLH26/54/66/138 (homologous with SCRM, SCREAM, also known as ICE1, Inducer of CBF Expression1) were predicted to collaborate with GubHLH133 (homolog of MUTE), GubHLH110 (homolog of FAMA), and GubHLH33/63 (homolog of SPCH, Speechless). The GubHLH106 (homolog of HEC1, HECATE1), GubHLH51/83 (homolog of HEC2), and GubHLH111 (homolog of HEC3) were predicted to interact with GubHLH23/84 (homolog of SPT, Spatula). GubHLH30 (homolog of AtbHLH100) was predicted to interact with GubHLH68/131 (homolog of FIT). GubHLH116 (homolog of MYC2), GubHLH108 (homolog of MYC3) and GubHLH109 (homolog of MYC4) were predicted to cluster into one functional module. The WD-repeat protein TTG1 (Transparent Testa Glabra1) was predicted to interact with TT8 (homolog of GubHLH38), GL3 (homolog of GubHLH64) and MYB75. TTG1, GL3, EGL3, and MYB75 have been reported to work in concert to modulate the accumulation of anthocyanin and initiation of trichome through JA signaling (Qi et al., 2011).

**Figure 6:**
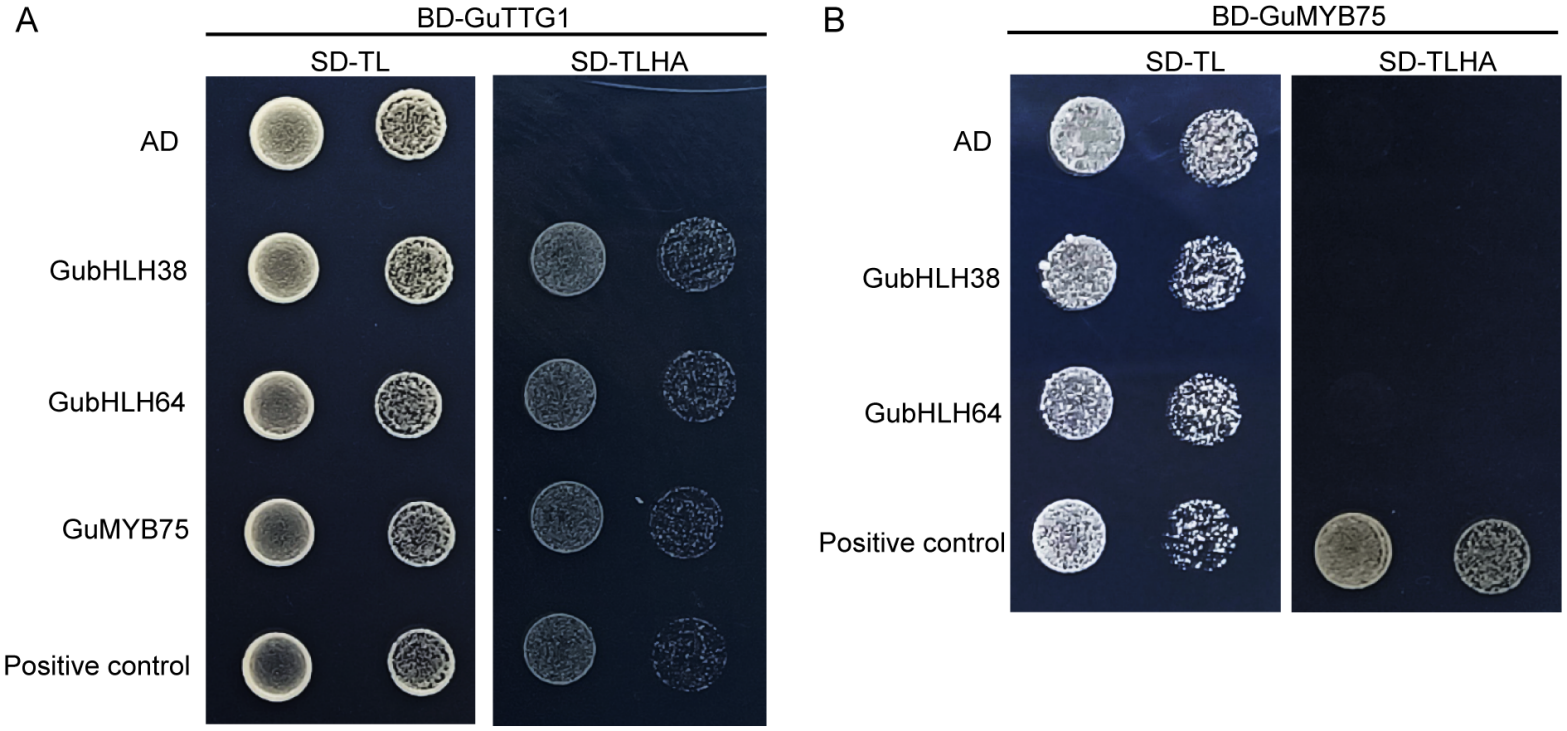
The Y2H assay of proteins in the anthocyanin biosynthesis and trichome formation associated network. **(A)** Interaction validation of GubHLH64, GubHLH38 and GuMYB75 with GuTTG1 via yeast two-hybrid assay. **(B)** Interaction validation of GubHLH38, GubHLH64 with GuMYB75 via yeast two-hybrid assay.

GubHLH64 interacted with GubHLH38, and GubHLH108, 109, 116 were clustered into one functional module according to the PPI network. In order to further explore their functions, subcellular localization assays of GubHLH38, 64, 108, 109, 116 were conducted using fluorescent reporter genes (GFP). The results showed that the fluorescence of *35S*::GubHLH38-GFP, *35S*::GubHLH64-GFP, *35S*::GubHLH108-GFP, *35S*::GubHLH109-GFP, and *35S*::GubHLH116-GFP was present in the nucleus of *N. benthamiana* (**Figure 5B**), suggesting that GubHLH38, 64, 108, 109, 116 are specifically localized to the nucleus. In present PPI network, TTG1 interacted with GL3 (homolog of GubHLH64), TT8 (homolog of GubHLH38) and MYB75. The yeast two-hybrid assays were performed to elucidate the potential interactions between GuTTG1, GubHLH64, GubHLH38 and GuMYB75 in *G. uralensis*, which are homologs of TTG1, GL3, TT8 and MYB75, respectively. These assays revealed that GubHLH38, GubHLH64, and GuMYB75 directly interact with GuTTG1 (**Figure 6A**). Whereas GuMYB75 was found not to interact directly with GubHLH38 and GubHLH64 (**Figure 6B**).

### 3.7 Expression profiles of *GubHLHs* under abiotic stress and phytohormone treatments and *cis*-acting elements analysis

**Figure 7:**
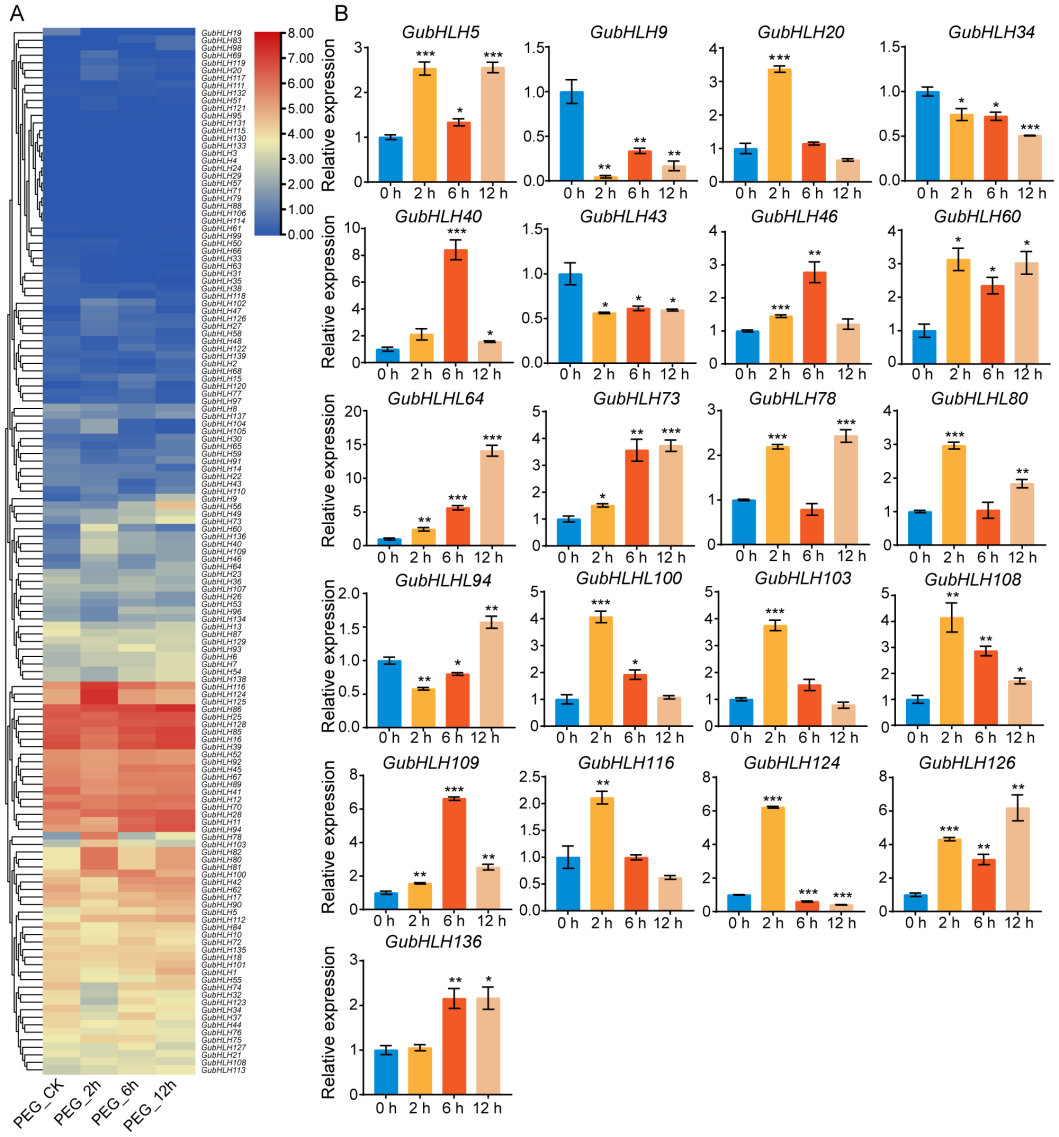
The expression profiles of *GubHLHs* under PEG treatments. **(A)** Heatmap of *GubHLHs* expression patterns under PEG treatment. **(B)** The selected *GubHLHs* expression levels in the licorice roots under PEG treatments via qRT-PCR. Student’s t-test was used to assess significant differences. Significance levels: *p < 0.05; **p < 0.01,***p < 0.001.

The expression profiles of *GubHLHs* under PEG treatment was analyzed according to the RNA-seq data (**Figure 7A**; **Supplementary Table S12**). *GubHLH47*, *GubHLH78*, *GubHLH60*, *GubHLH126*, *GubHLH136*, *GubHLH80*, *GubHLH40*, *GubHLH46*, *GubHLH124*, *GubHLH103*, *GubHLH116*, *GubHLH64*, *GubHLH112*, and *GubHLH100* were obviously increased by PEG at 2 h. Conversely, *GubHLH13*, *GubHLH32*, *GubHLH34*, *GubHLH123*, and *GubHLH74* were downregulated at 2 h. *GubHLH47*, *GubHLH120*, *GubHLH9*, *GubHLH136*, *GubHLH73*, *GubHLH60*, *GubHLH56*, *GubHLH40*, *GubHLH100*, *GubHLH112*, and *GubHLH94* showed increased expression at 6 h. *GubHLH9*, *GubHLH56*, *GubHLH73*, *GubHLH78*, *GubHLH64*, *GubHLH46*, *GubHLH49*, *GubHLH136*, *GubHLH94*, *GubHLH112*, *GubHLH80*, and *GubHLH5* were upregulated at 12 h. Furthermore, qRT-PCR was performed on 21 selected *GubHLHs* to validate their expressions under PEG treatment (**Figure 7B**). Most of the *GubHLHs* exhibited similar expression patterns in the RNA-seq and qRT-PCR analyses. *GubHLH103*, *GubHLH116*, *GubHLH124*, and *GubHLH20* were induced by 3.75, 2.11, 6.21, and 3.37 folds at 2 h, respectively. *GubHLH40* was induced significantly by 8.41 folds after 6 *h* treatment*. GubHLH136* was up to 2.16 fold after 6 *h* and 12 h treatment, respectively. *GubHLH5* showed inductions of 2.53, 1.34, and 2.56 folds at 2, 6, and 12 h. *GubHLH64* exhibited increased expressions by 2.44, 5.63, and 14.08 folds at 2, 6, and 12 h. *GubHLH60*, *GubHLH73*, *GubHLH108*, *GubHLH109*, and *GubHLH126* were also upregulated at three time points. *GubHLH46* and *GubHLH100* were markedly induced after treatment of 2 h and 6 h. *GubHLH78* and *GubHLH80* were significantly upregulated at 2 h and 12 h, respectively.

**Figure 8:**
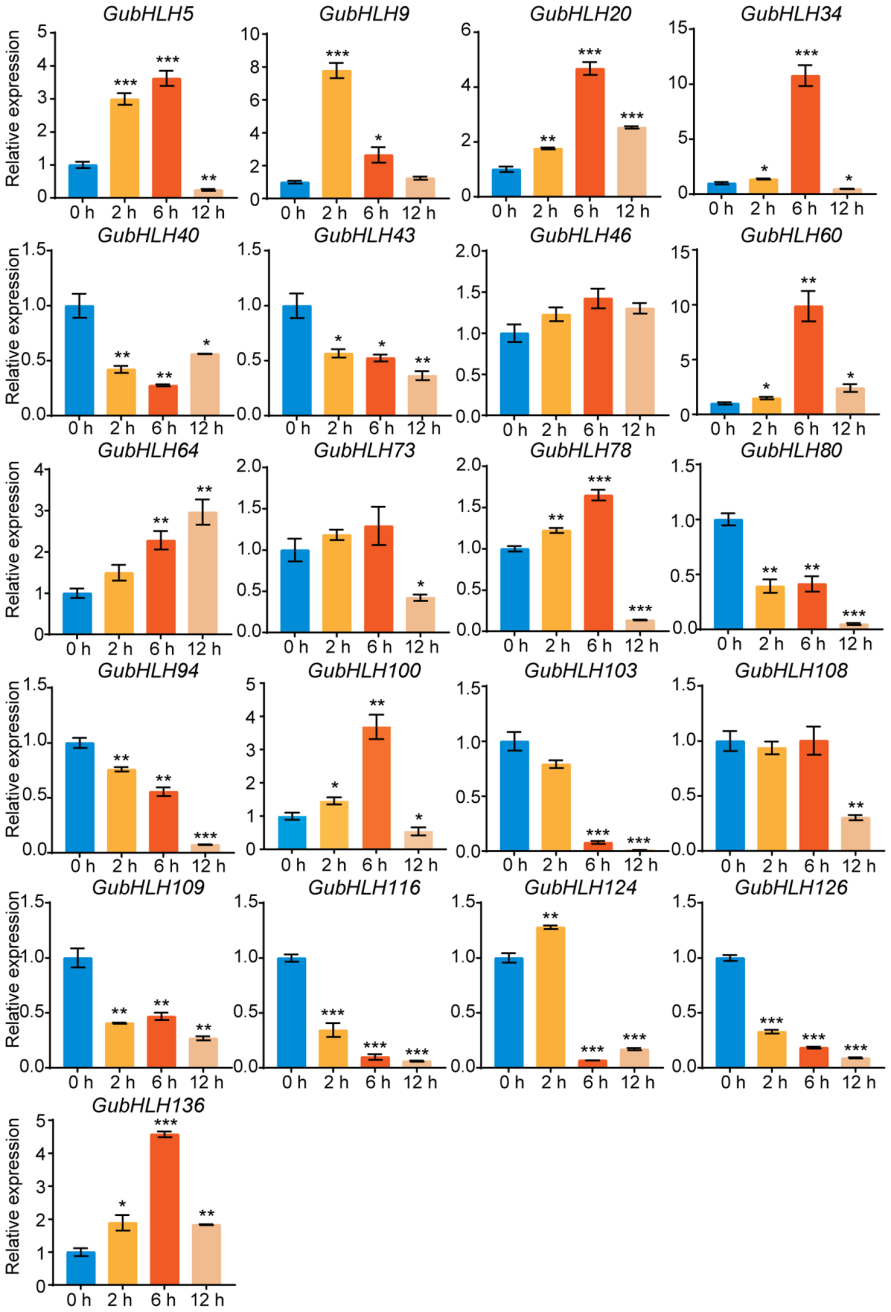
The selected *GubHLHs* expression levels in the licorice roots under NaCl treatments via qRT-PCR. Significant differences were evaluated by student’s t-test. Significance levels: *p < 0.05; **p < 0.01,***p < 0.001.

Moreover, the expression of these genes in licorice roots under NaCl treatment was also investigated via qRT-PCR (**Figure 8**). The results showed that *GubHLH40*, *GubHLH43*, *GubHLH80*, *GubHLH94*, *GubHLH103*, *GubHLH109*, *GubHLH116*, and *GubHLH126* were reduced by NaCl. *GubHLH5*, *GubHLH34*, *GubHLH78*, and *GubHLH100* showed increased expression trends after 2 h and 6 h of treatments, followed by a decrease at 12 h, with the highest expression occurring at 6 h, with 3.62-, 10.79-, 1.65-, and 3.69-fold increases, respectively. *GubHLH9* was induced by 7.78 and 2.65 folds after 2 h and 6 h NaCl treatment, respectively. *GubHLH20*, *GubHLH60*, and *GubHLH136* were induced at three time points. The expression of *GubHLH124* was increased to 1.28 folds at 2 h. *GubHLH64* exhibited the increase of 2.28 and 2.97 folds at 6 h and 12 h, respectively.

**Figure 9:**
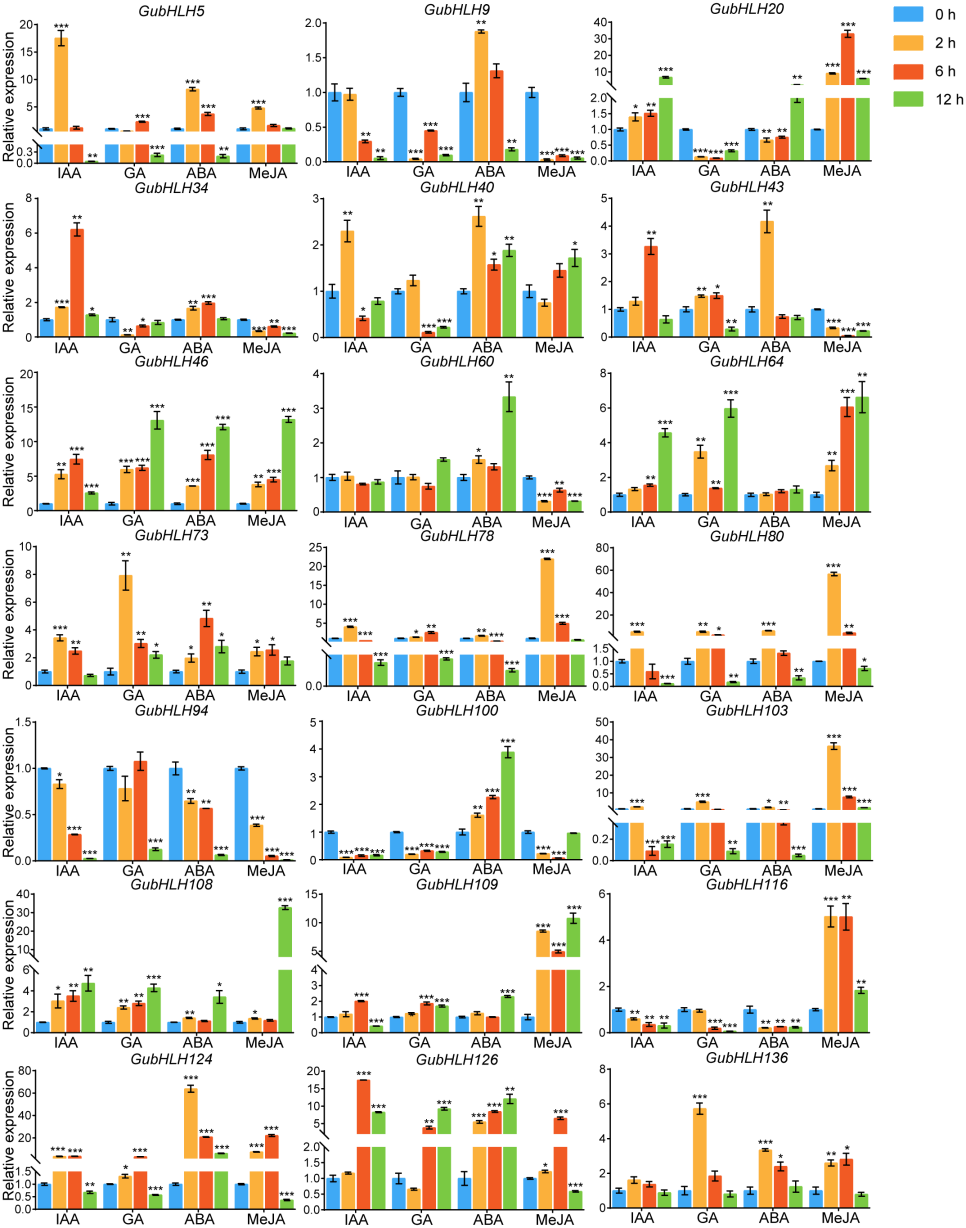
Expression profiles of *GubHLHs* in response to IAA, MeJA, ABA and GA treatments based on qRT-PCR analysis. Samples were collected at 0 h, 2 h, 6 h and 12 h after the four kinds of hormone treatments. Significant differences were estimated through student’s t-test. Significance levels: *p < 0.05; **p < 0.01,***p < 0.001.

Phytohormones play substantial roles in plant growth, development and defense. We further investigated the response of these *GubHLHs* to four kinds of phytohormones, namely, IAA, GA, ABA, and MeJA, via qRT-PCR, and multifarious temporal-spatial expression profiles were acquired (**Figure 9**). Under IAA treatment, *GubHLH5*, *GubHLH40*, *GubHLH73*, *GubHLH78*, *GubHLH80*, and *GubHLH103* reached the highest expression at 2 h, with increases of 17.55, 2.30, 3.44, 4.05, 5.10, and 2.12 folds, respectively. The expressions of *GubHLH34*, *GubHLH43*, *GubHLH46*, *GubHLH109*, *GubHLH124*, and *GubHLH126* peaked at 6 h, showing increases of 6.21, 3.27, 7.45, 2.01, 3.63, and 17.48 folds, respectively. *GubHLH20*, *GubHLH64* and *GubHLH108* expressions were 6.73, 4.57 and 4.73 folds higher after being treated for 12 h, respectively. *GubHLH9*, *GubHLH100*, and *GubHLH116* were reduced by IAA. Under GA treatment, *GubHLH9*, *GubHLH20*, *GubHLH34*, *GubHLH100*, and *GubHLH116* exhibited downregulation. *GubHLH43*, *GubHLH78*, *GubHLH80*, and *GubHLH124* showed increases at 2 and 6 h. *GubHLH46*, *GubHLH64*, *GubHLH73*, and *GubHLH108* were upregulated at all three time points. *GubHLH109* and *GubHLH126* were induced at 6 h and 12 h. *GubHLH5* was induced at 6 h, whereas *GubHLH103* and *GubHLH136* were induced at 2 h. Under ABA treatment, *GubHLH9*, *GubHLH43*, *GubHLH78*, *GubHLH80*, and *GubHLH103* were induced by 1.88, 4.17, 1.68, 5.99, and 1.80 folds at 2 h, respectively. *GubHLH20*, *GubHLH60*, and *GubHLH109* were up to 2.08, 3.33 and 2.31 folds at 12 h, respectively. *GubHLH40*, *GubHLH46*, *GubHLH73*, *GubHLH100*, *GubHLH124*, and *GubHLH126* were upregulated at 2, 6, and 12 h. *GubHLH5*, *GubHLH34*, and *GubHLH136* exhibited increases at 2 h and 6 h. *GubHLH124* showed the highest expression after being treated for 2 h with a 63.91-fold rise. *GubHLH94* and *GubHLH116* expressions were reduced by ABA. Regarding the MeJA treatment, *GubHLH9*, *GubHLH34*, *GubHLH43*, *GubHLH60*, and *GubHLH94* were significantly reduced by MeJA. The expression of *GubHLH5* was increased by 4.79 folds after 2 h of treatment. *GubHLH40* was induced by 1.72 folds after 12 h of treatment. The expression of *GubHLH73*, *GubHLH78*, *GubHLH80*, *GubHLH124*, *GubHLH126*, and *GubHLH136* were upregulated at 2 h and 6 h. Among them, *GubHLH78* and *GubHLH80* showed the greatest increase at 2 h and reached 21.98 and 56.67 folds higher levels, respectively, than those in the control. *GubHLH73*, *GubHLH124*, *GubHLH126*, and *GubHLH136* were induced by 2.56, 22.12, 6.52, and 2.81 folds at 6 h. *GubHLH20*, *GubHLH46*, *GubHLH64*, *GubHLH103*, *GubHLH108*, *GubHLH109*, and *GubHLH116* were induced at all three time points. Among them, the expression levels of *GubHLH103* and *GubHLH116* peaked at 2 h, and were 36.44 and 5.02 folds higher than those in the control, respectively. The expression of *GubHLH20* peaked at 6 h and was upregulated 33.04 folds. The expression of *GubHLH46*, *GubHLH64*, *GubHLH108*, and *GubHLH109* peaked at 12 h, with increases of 13.20, 6.63, 32.70 and 10.78 folds, respectively. These analyses demonstrated the presumable function of these *GubHLHs* in *G. uralensis* phytohormone responses.

Promoter *cis*-element analysis always contribute to the understanding of gene regulation mechanisms and expression profiles. The 2000 bp upstream promoter sequences of *GubHLHs* were extracted for *cis*-acting elements analysis. The results revealed the presence of 19089 *cis*-acting elements in their promoters (**Supplementary Table S13**). Among them, a variety of *cis*-acting elements associated with stress responses (2571, 13.47%), phytohormone responses (1325, 6.94%) and plant growth and development (251, 1.31%) were identified (**Supplementary Figure S7**). The abiotic/biotic stress related *cis*-acting elements were found in all *GubHLH* promoters. Among them, MYB elements were the most prevalent, with 624 instances, followed by MYC (548), STRE (383), ARE (256), as-1 (170), W box (104), WUN-motif (100), WRE3 (93), MBS (92), TC-rich repeats (63), LTR (60), MYB recognition site (42), GC-motif (26), and MBSI (10) (**Supplementary Figures S7A, B**). The MBS elements involved in drought-inducibility were identified in the promoters of 64 *GubHLHs* including *GubHLH9*, *GubHLH20*, *GubHLH34*, *GubHLH40*, *GubHLH47*, *GubHLH73*, *GubHLH94*, *GubHLH112*, *GubHLH123* and *GubHLH126*, whose responses to drought might be related to these elements. And the phytohormone responsive related *cis*-elements were also found in these *GubHLHs* promoters. The IAA responsive elements (52 TGA-element, 19 AuxRR-core) were predicted in 54 *GubHLHs*. And the response of *GubHLH5*, *GubHLH34*, *GubHLH40*, *GubHLH73*, *GubHLH78*, *GubHLH80*, *GubHLH109* and *GubHLH116* to IAA might be related to the IAA responsive elements. Eighty-one GubHLHs contained gibberellin responsive elements (62 P-box, 34 GARE-motif, 29 TATC-box). The GA responses of *GubHLH5*, *GubHLH40*, *GubHLH43*, *GubHLH46*, *GubHLH64*, *GubHLH73*, *GubHLH94* and *GubHLH136* might be relevant to these elements. ABA responsive elements (365 ABRE, 66 ABRE3a, 66 ABRE4) were presented in promoters of 112 *GubHLH* genes. The expressions of *GubHLH5*, *GubHLH9*, *GubHLH34*, *GubHLH40*, *GubHLH43*, *GubHLH46*, *GubHLH60*, *GubHLH73*, *GubHLH78*, *GubHLH80*, *GubHLH94*, *GubHLH100*, *GubHLH103*, *GubHLH109*, *GubHLH116*, *GubHLH124* and *GubHLH136* were increased or decreased significantly under ABA treatment. Moreover, 170 TGACG-motif and 170 CGTCA-motif which are involved in MeJA response were distributed in the promoters of 88 *GubHLHs*. *GubHLH5*, *GubHLH9*, *GubHLH34*, *GubHLH40*, *GubHLH43*, *GubHLH46*, *GubHLH60*, *GubHLH73*, *GubHLH80*, *GubHLH94*, *GubHLH100*, *GubHLH109*, *GubHLH124* and *GubHLH136* were induced or reduced by MeJA. Additionally, the *cis*-acting elements involved in circadian regulation (31 circadian), seed-specific regulation (13 RY-element), meristem expression (68 CAT-box, 18 CCGTCC-box), zein metabolism regulation (81 O2-site), differentiation of palisade mesophyll cells (9 HD-Zip 1), and endosperm expression (31 GCN4_motif) were also identified.

## 4 Discussion

The bHLH TFs represent one of the maximum TF families and are engaged in a wide array of growth, development, and environmental adaptation processes. The expeditious advancement of plant genome sequencing technology has facilitated the studies of plant bHLH gene families. Identifying various bHLH isoforms and investigating their expression patterns are fundamental to comprehend their functions. Nonetheless, limited research has been conducted on the medicinal plant licorice, which garners significant attention for its medicinal and industrial applications. In present study, a systematic and comprehensive investigation of *G. uralensis* bHLH family members was undertaken. A total of 139 genes encoding *G. uralensis* bHLH TFs were identified, with protein lengths varying from 73 to 777 amino acids and pI ranging from 4.55 to 10.29, suggesting potential structural and functional diversity. And 138 out of 139 GubHLH proteins were predicted to localize in the nucleus, emphasizing their primary roles in regulation and interaction within the nucleus. This localization pattern was generally in accord with the GO enrichment results, where the majority of GubHLHs were enriched in the nucleus at the cellular component level. Multiple alignment analysis revealed four conserved regions within the bHLH domain, aligning with the characteristics of bHLH TFs (Atchley et al., 1999; Nair and Burley, 2000). A total of 23 conserved amino acids were found with a consensus ratio greater than 50%, and seven out of these showed a consensus ratio greater than 75%. These findings are consistent with previous studies (Li et al., 2006; Zhang et al., 2018). The extremely conserved Leu-23 and Pro-28, respectively accounting for 98.6% and 97.1%, underscored their significance in dimerization formation (Atchley et al., 1999). And in the basic region, the conserved H_5_-E_9_-R_13_ region was present in GubHLH proteins, being in line with the characteristic of most plants (Heim et al., 2003; Qian et al., 2007; Pires and Dolan, 2010). A total of 403 G-box *cis*-acting elements were identified in the *GubHLH* gene promoters and *GubHLH23* possessed the highest number of 19 of them. And 624 MYB *cis*-acting elements were detected that were existed in all *GubHLH* gene promoters. These results indicated that these GubHLH TFs may function through interacting with MYB TFs or among themselves, as also supported by the GO annotations of GubHLH TFs with binding activity.

The plant *bHLH* TF family comprises approximately 14-32 subfamilies (Heim et al., 2003; Toledo-Ortiz et al., 2003; Li et al., 2006; Carretero-Paulet et al., 2010; Pires and Dolan, 2010). Phylogenetic analysis classified GubHLHs into 25 subfamilies, with varying subfamily sizes ranging from 1 to 16 members. And it was shown that some gene pairs exhibited one-to-one orthologous relationships, such as AtbHLH71 and GubHLH117 in subfamily Ia, AtbHLH142 and GubHLH6 in subfamily XIV, AtbHLH95 and GubHLH71 in subfamily Ib (1), AtbHLH102 and GubHLH89 in subfamily Va, as well as AtbHLH108 and GubHLH14 in subfamily VI. In more cases, bHLH proteins from the same species were inclined to cluster together. Moreover, phylogenetic tree indicated that subfamily IVa contained six pairs of duplication *GubHLHs*, including three segmental duplication gene pairs (*GubHLH73*/*136*, *GubHLH80*/*103*, *GubHLH78*/*120*) and three tandem duplication gene pairs (*GubHLH80*/*81*, *GubHLH103*/*104*, *GubHLH122*/*123*). Subfamily Vb contained five pairs of segmental duplication *GubHLHs* (*GubHLH87*/*93*, *GubHLH62*/*96*, *GubHLH72*/*129*, *GubHLH8*/*91*, *GubHLH31*/*34*). These results indicated that species-specific duplication events are of significant importance in gene family evolution. And the specific genes might play significant roles in licorice*. GubHLH20*, *GubHLH73*, *GubHLH78*, *GubHLH80* and *GubHLH136* in subfamily IVa showed obvious up-regulated or down-regulated trends under PEG, NaCl and four phytohormones treatments, indicating that these GubHLHs play roles in the abiotic and phytohormones responses. Their regulatory mechanisms and functions should be further confirmed. MEME analysis revealed the presence of conserved motifs crucial for the structure and function specificity of bHLH domain in all GubHLH proteins (Feller et al., 2011). The varied exon/intron structures of the members within gene families are notable clues to apprehend their evolution and diversified functions, which are achieved via three main ways, insertion/deletion, exon/intron gain/loss, and exonization/pseudoexonization (Xu et al., 2012). A varying number of introns ranging from 0 to 19 was revealed in *GubHLHs*. which was similar to apple *bHLH* genes (Yang et al., 2017). It may be concluded that exon/intron gain/loss occurred during the evolution process of the *G. uralensis* bHLH family. With 15 UTRs, *GubHLH138* may own the most complicated alternatively spliced form, and similar results were also obtained in passion fruit (Liang et al., 2023). These results indicated that closely related bHLH members are inclined to exhibit similar exon/intron structures, motif compositions and secondary protein structures. Gene duplication events are indispensable for the gene family expansion in the evolutionary process and the new members and functions generation (Flagel and Wendel, 2009). Segmental duplication played a prominent role, resulting in 55 pairs of segmentally duplicated genes. Forty-one pairs of these genes owned differential gene structures. For instance, *GubHLH83* contained one exon, while its paralog, *GubHLH106*, had four exons, indicating the acquisition of three exons during evolutionary progression, a phenomenon similarly observed in *Raphanus sativus* and passion fruit (Wang et al., 2022; Liang et al., 2023). The purifying selection is highlighted by the Ka/Ks values exceeding 1 for all duplicated gene pairs, reflecting selective pressure throughout *GubHLH* genes evolution. This observation aligns with findings in other species, including *Brassica oleracea*, *Brassica napus*, and *Brassica rapa* (Miao et al., 2020), and rice (Li et al., 2006). Comparative analyses with other plant species demonstrated a higher number of collinear pairs between *G. uralensis* and *G. max*, and between *G. uralensis* and *M. truncatula*, indicative of their closer evolutionary connections.

At present, environmental stresses such as drought and salt stress have been the influencing factors of the plant progression and ecological environment (Liu et al., 2014). Expression pattern analysis is conducive to understanding gene function and characteristics. bHLH TFs were indicated to be engaged in regulating abiotic stress tolerance. *AtbHLH112* transcript levels were shown to be positively related to PEG and NaCl tolerance, enhancing stress tolerance (Liu et al., 2015). AtbHLH122 was indicated to play a notable role in positively regulating drought, NaCl and osmotic resistance (Liu et al., 2014). The rd22BP1 (AtbHLH6) protein is significantly induced by dehydration, and ABA can activate the transcription of the dehydration-responsive *rd22* gene (Abe et al., 1997). The *AtbHLH17* (*AtAIB*) expression was markedly increased under drought stress and ABA treatment, and the coexpression of AtbHLH17 with *AtWRKY28* can strengthen the transcription of downstream stress-responsive genes, in turn, enhance stress tolerance (Babitha et al., 2013). MfbHLH38 was shown to heighten drought and salinity resistance and to be involved in the ABA-dependent pathway (Qiu et al., 2020). Several licorice *bHLH*s were induced or reduced by PEG treatment, which might indicate their involvement in regulating the PEG response. *GubHLH40*, *GubHLH46*, *GubHLH60*, *GubHLH108*, *GubHLH109*, and *GubHLH116*, which clustered into subfamily III (d+e) with *AtbHLH6* and *AtbHLH17*, showed increased trends after PEG treatment in both RNA-seq and qRT-PCR data. Moreover, *GubHLH100*, which belonged to subfamily XI along with *AtbHLH122*, was induced obviously by PEG treatment. These results might indicate the potential involvement of these genes in the modulation of drought tolerance and the improvement of adaptability to the environment. Additionally, ABA accumulation is necessary for the expression inductions of some gene under drought stress (Zhu, 2002). In our study, the expressions of *GubHLH40*, *GubHLH46*, *GubHLH60*, *GubHLH108*, and *GubHLH109* tended to increase under ABA treatment, coinciding with the findings for *AtbHLH17* and *AtbHLH6*. Whereas the *GubHLH116* was reduced by ABA, which requires further in-depth exploration. In addition, significant induction of *GubHLH64* expression was detected upon both PEG and NaCl treatments, which might show the involvement of *GubHLH64* in licorice drought and salt stress.

The sophisticated interactions between GubHLH proteins and other proteins potentially enable their involvement in regulating multiple biological processes. Typically, three types of TFs, WD-repeat, bHLH, and MYB, can form complexes and modulate the anthocyanin accumulation and trichome development in a series of plants (Qi et al., 2011). The *Arabidopsis* WD40 repeat protein TTG1 occupies a central position in this complex, serving as a scaffold for the interactions between the other two TFs (Ramsay and Glover, 2005). The *Medicago truncatula* bHLH TF MtTT8 can form a complex with MtWD40-1 and MtLAP1 or MtPAR, regulating the synthesis of anthocyanins and proanthocyanidin through activating the downstream anthocyanidin reductase (MtANS) and anthocyanidin synthase (MtANR) (Li P. et al., 2016). In *Prunus avium*, PavWD40, PavMYB10.1, and PavbHLH interact with each other and regulate the cherry anthocyanin biosynthesis (Jin et al., 2016). JA, a significant plant hormone, mediates multifarious developmental processes in plants, functioning as a regulatory molecule (Li et al., 2021). JA has been previously reported to favor the anthocyanin accumulation (Shan et al., 2009) and trichome initiation (Traw and Bergelson, 2003). *Arabidopsis* GL3, TT8 and EGL3 have been shown to interact with MYB75 and TTG1, forming WD-repeat/bHLH/MYB complexes and modulating the accumulation of anthocyanin and initiation of trichome through JA signaling (Qi et al., 2011). In present PPI network, TT8, homologous with GubHLH38, and GL3 (homolog of GubHLH64), along with TTG1 and MYB75, were clustered into an interconnected functional module. Phylogenetic analysis showed that GubHLH64 and GubHLH38 are clustered into the same branch (subfamily IIIf) together with GL3, TT8 and EGL3, indicating that these bHLHs may exert similar functions. Subcellular localization assays confirmed that GubHLH64 and GubHLH38 are localized in nucleus. The expression of *GubHLH64* was prominently induced by MeJA. Yeast two-hybrid assays verified that GubHLH38, GubHLH64, and GuMYB75 directly interact with GuTTG1. These results indicate that GubHLH64 and GubHLH38 might be closely relevant to the regulation of trichome initiation and anthocyanin biosynthesis in *G. uralensis*. And their precise regulatory mechanisms and functions should be further confirmed.

MYC2, MYC3 and MYC4 can form homo- and heterodimers among themselves and also interact with JAZ proteins, playing a prominent role in activating the JA regulated transcriptional response (Fernández-Calvo et al., 2011). MYC2/MYC3/MYC4 were also found to be able to originate a hierarchical network of a series of downstream TFs, establishing a core MYC2/MYC3/MYC4-dependent “regulon” (Van Moerkercke et al., 2019). In the PPI network, MYC2 (homolog of GubHLH116), MYC3 (homolog of GubHLH108), and MYC4 (homolog of GubHLH109) were clustered into one functional module, forming a closely interconnected cluster. Further investigation into the subcellular localization of GubHLH108, GubHLH109, and GubHLH116 confirmed their presence in the nucleus, aligning with their roles as TFs. The expression of *GubHLH108*, *GubHLH109*, and *GubHLH116* were found to increase significantly after MeJA treatment. These results might imply the noteworthy functions of these three GubHLHs in a JA-regulated manner.

## 5 Conclusion

A total of 139 *G. uralensis bHLH* genes were identified and categorized into 25 subfamilies on the basis of the phylogenetic relationships with *Arabidopsis bHLH* genes. All GubHLH proteins exhibited the typical conserved bHLH domain, comprising four conserved regions with 23 amino acid residues. The analysis of motifs and domain composition, in conjunction with gene structure assessment, illustrated the relative conservation within specific subfamilies. Collinearity analysis emphasized the predominant contribution of segmental duplication to the expansion of *GubHLH* gene family, which experienced purifying selection throughout its evolution. The divergent exon/intron structures, observed in some of the duplication gene pairs might clarify their function diversification in evolution. The temporospatial expression profiles of GubHLHs following treatment with PEG, NaCl, and various phytohormones demonstrated their involvement in regulation of abiotic and phytohormones responses. GubHLH64 and GubHLH38 might be closely pertinent to the *G. uralensis* trichome initiation and anthocyanin biosynthesis, and GubHLH64 also related to the abiotic responses such as drought and salt. Several bHLH members in subfamily III (d+e) might participate in the *G. uralensis* drought response. GubHLH108, GubHLH109, and GubHLH116 might exploit noteworthy functions in a JA-dependent manner. The results gained in current study can favor the deeper insight into the characteristics, evolution, and expression patterns of GubHLH proteins, offering a foundation for future GubHLHs biological function explorations.

## Data Availability

The original contributions presented in the study are included in the article/**Supplementary Material**. Further inquiries can be directed to the corresponding authors.
