# Supplementary material for: Genome-wide identification and expression analysis of bHLH gene family revealed their potential roles in abiotic stress response, anthocyanin biosynthesis and trichome formation in Glycyrrhiza uralensis
Source: Front Plant Sci. 2025 Jan 21;15:1485757. doi: 10.3389/fpls.2024.1485757 (PMC11790457; doi:10.3389/fpls.2024.1485757)
Supplement: Supplementary file 1 [file SupplementaryFile1.docx]

Supplementary Figures


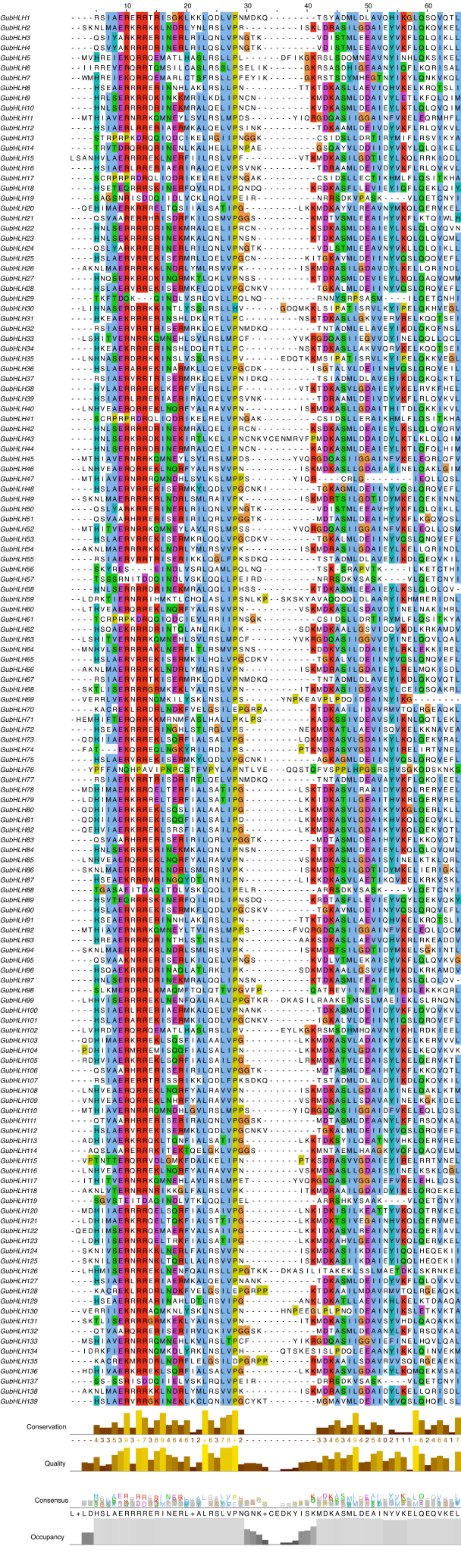


Figure S1 The multiple amino acids sequence alignment analysis of bHLH domain in GubHLH proteins.


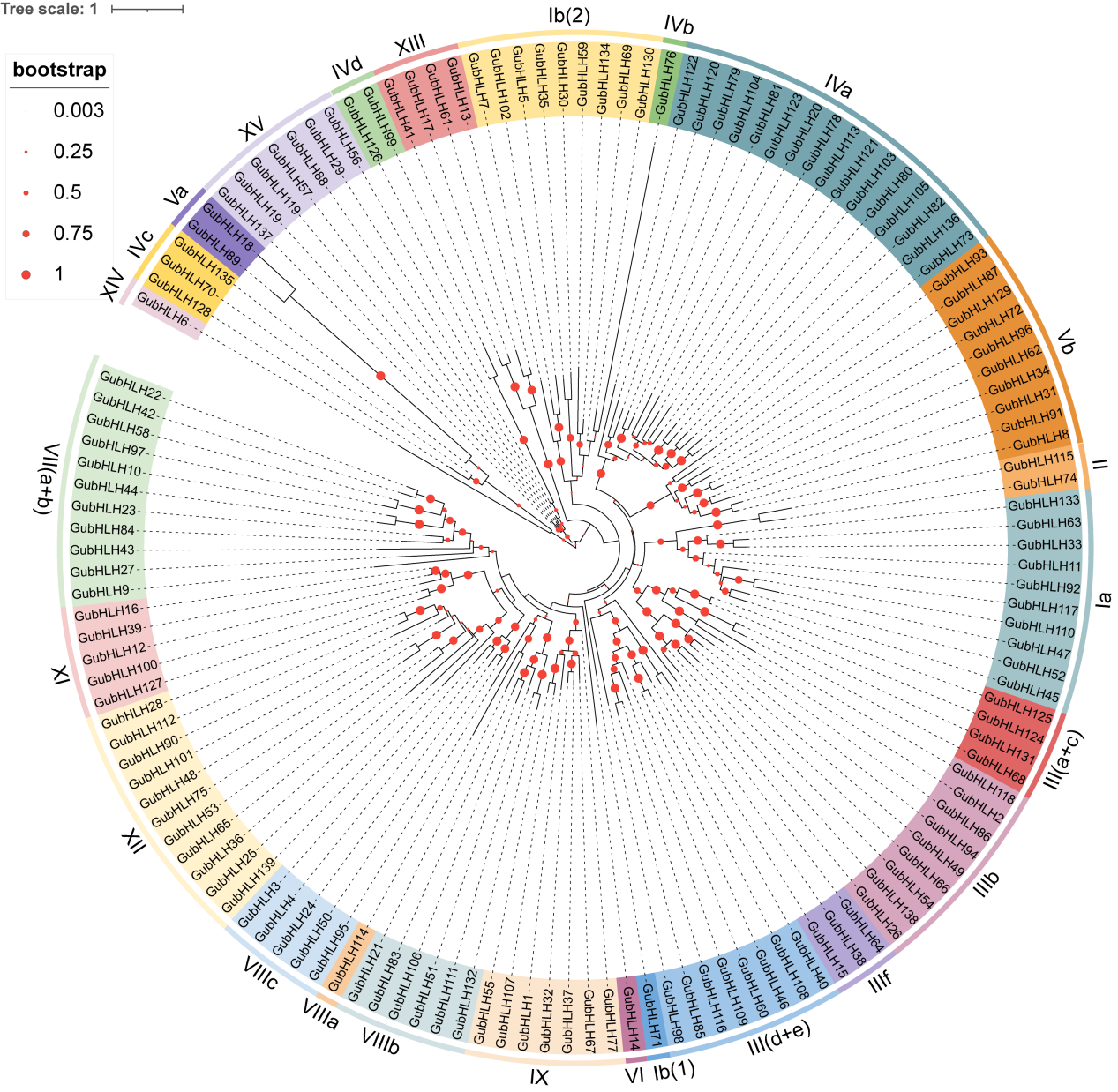


Figure S2 The phylogenetic tree of bHLH family members in *G. uralensis*. The GubHLH protein subgroups were presented by differently coloured branches.


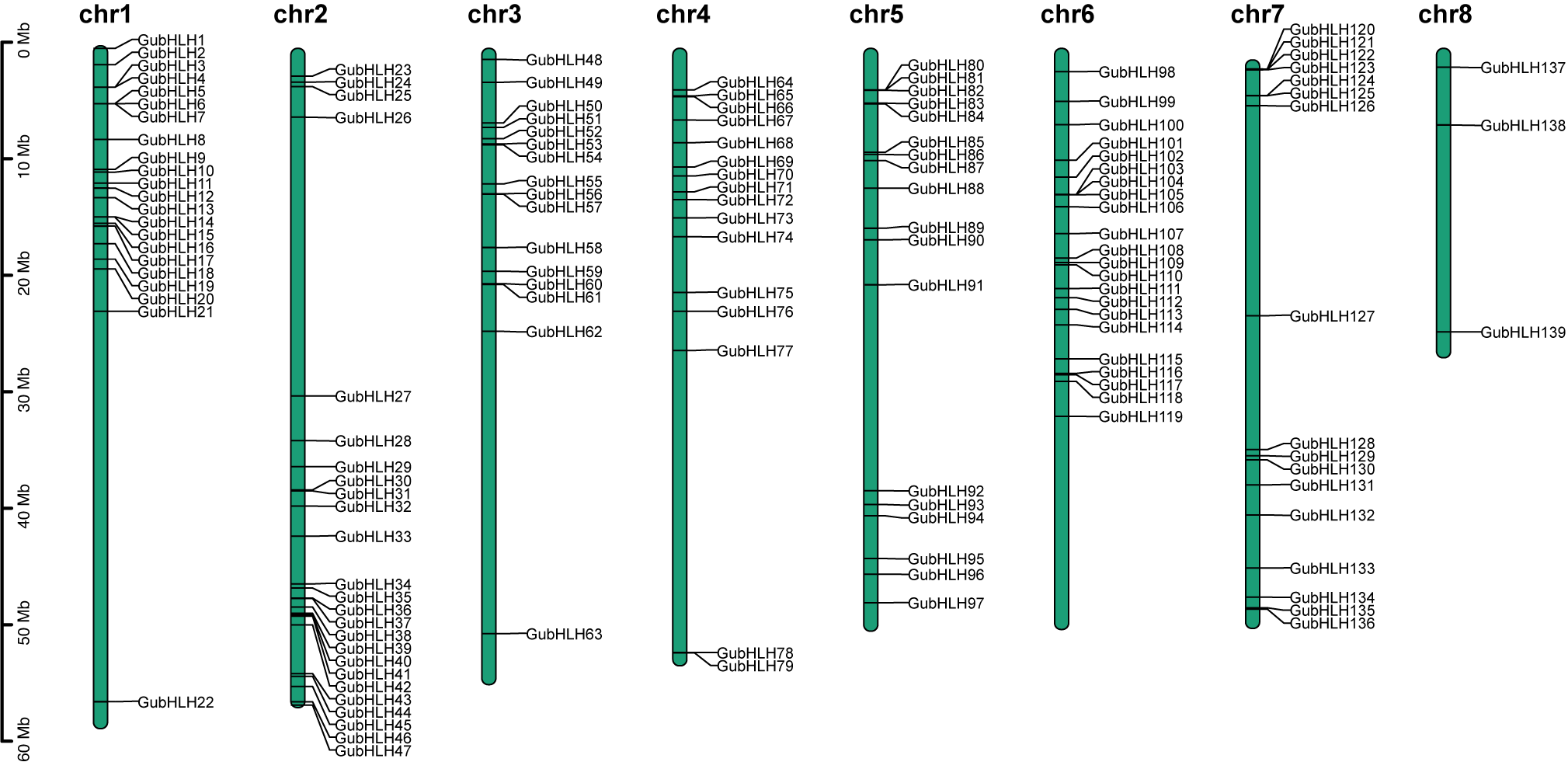


Figure S3 The chromosomal locations of the *G. uralensis bHLH* genes. The vertical bar on the left side represented the length of chromosome. Mb, megabase.


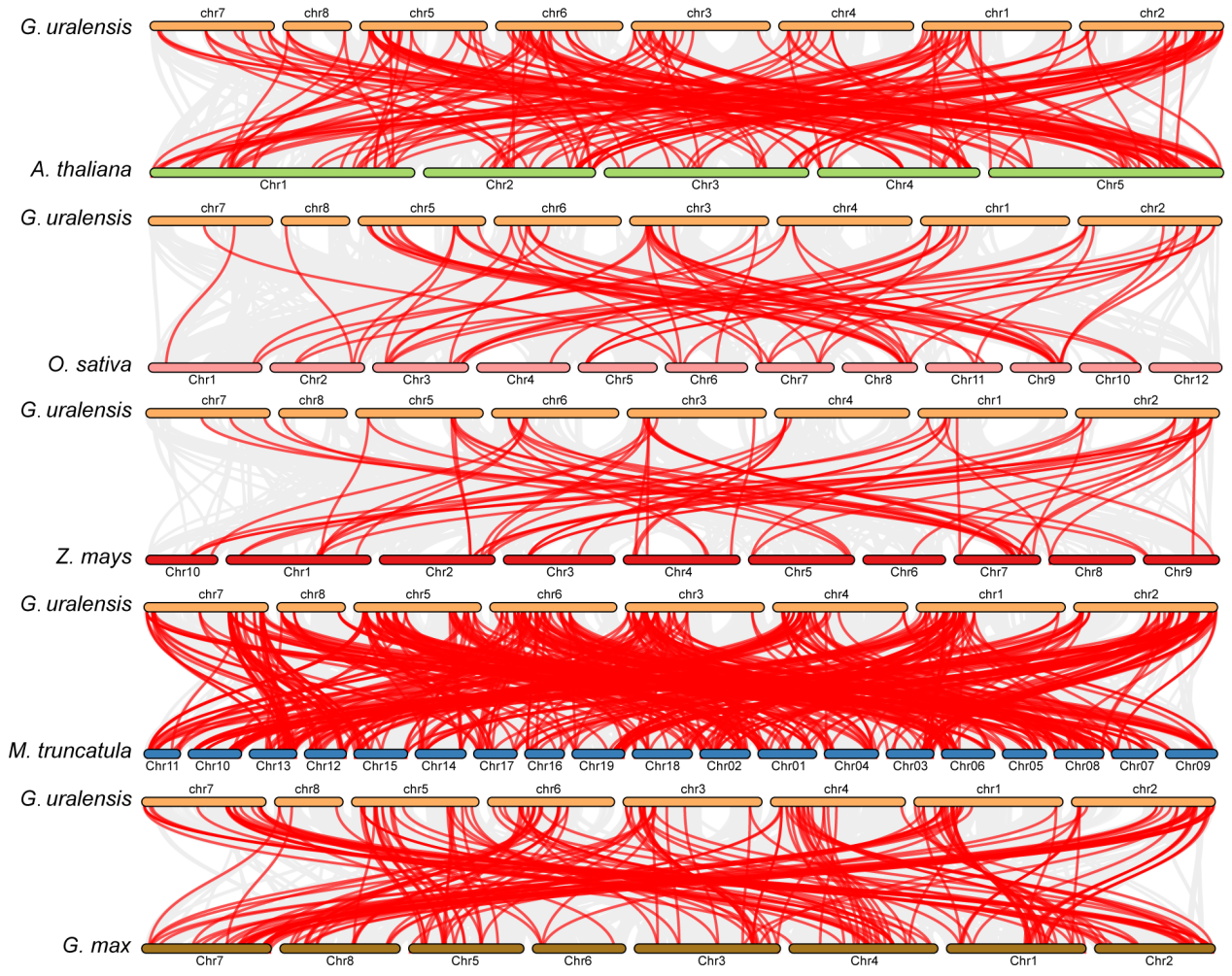


Figure S4 Synteny analysis of *bHLHs* between licorice and other five plants. The grey lines indicate the collinear blocks within licorice and other plant genomes, and the red lines highlights the collinear relationships within *GubHLH* genes, the numbers represent the chromosome numbers of plant genomes. Species names are displayed with “*A. thaliana*”, “*O. sativa*”, “*Z. mays*”, “*M. truncatula*”, and “*G. max*” for *Arabidopsis thaliana*, *Oryza sativa*, *Zea mays*, *Medicago truncatula* and *Glycine max*, respectively.


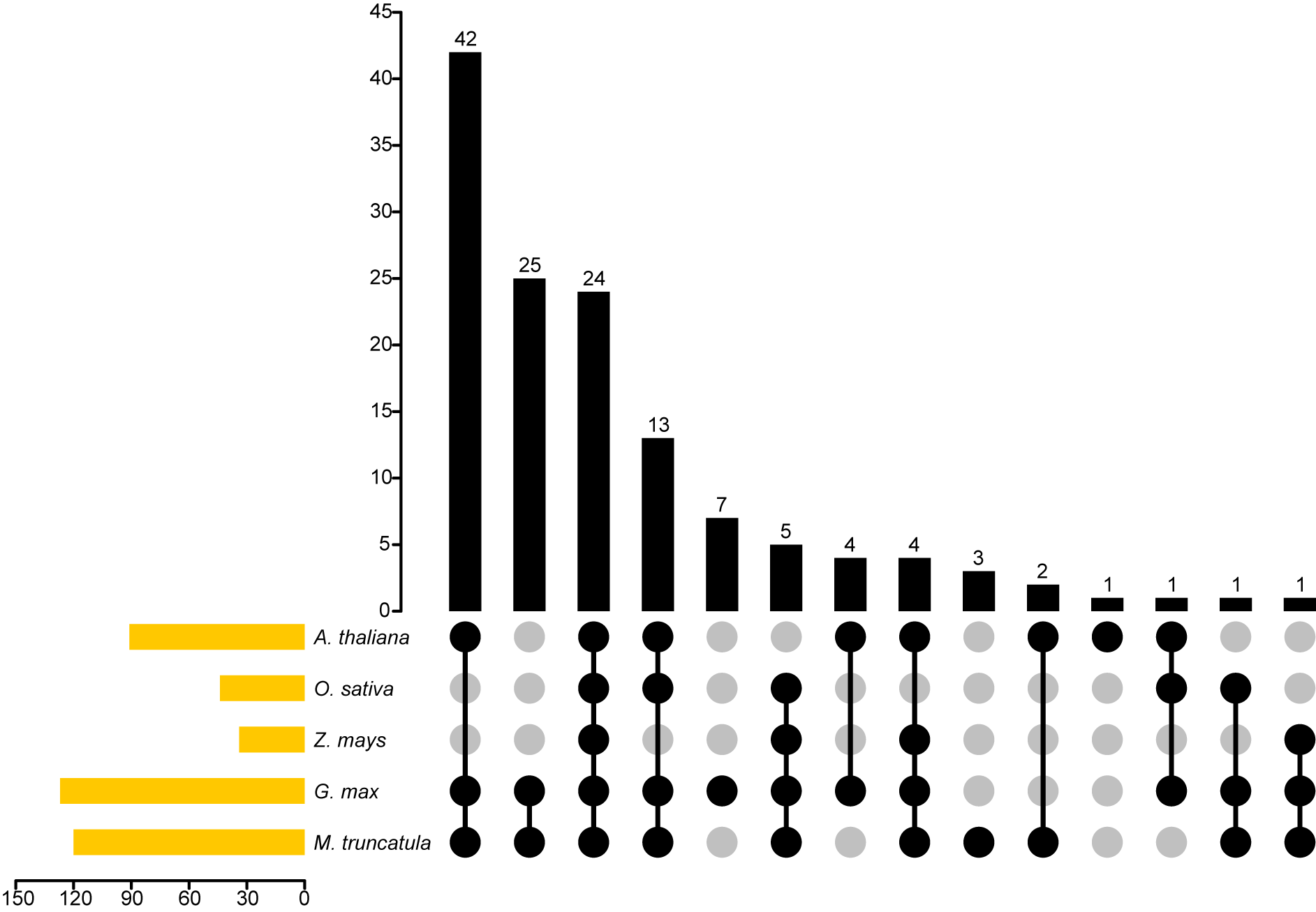


Figure S5 UpSet diagram illumining the numbers of shared GubHLHs exhibited collinear associations with five representative species. Species names are displayed with “*A. thaliana*”, “*O. sativa*”, “*Z. mays*”, “*M. truncatula*”, and “*G. max*” for *Arabidopsis thaliana*, *Oryza sativa*, *Zea mays*, *Medicago truncatula* and *Glycine max*, respectively.


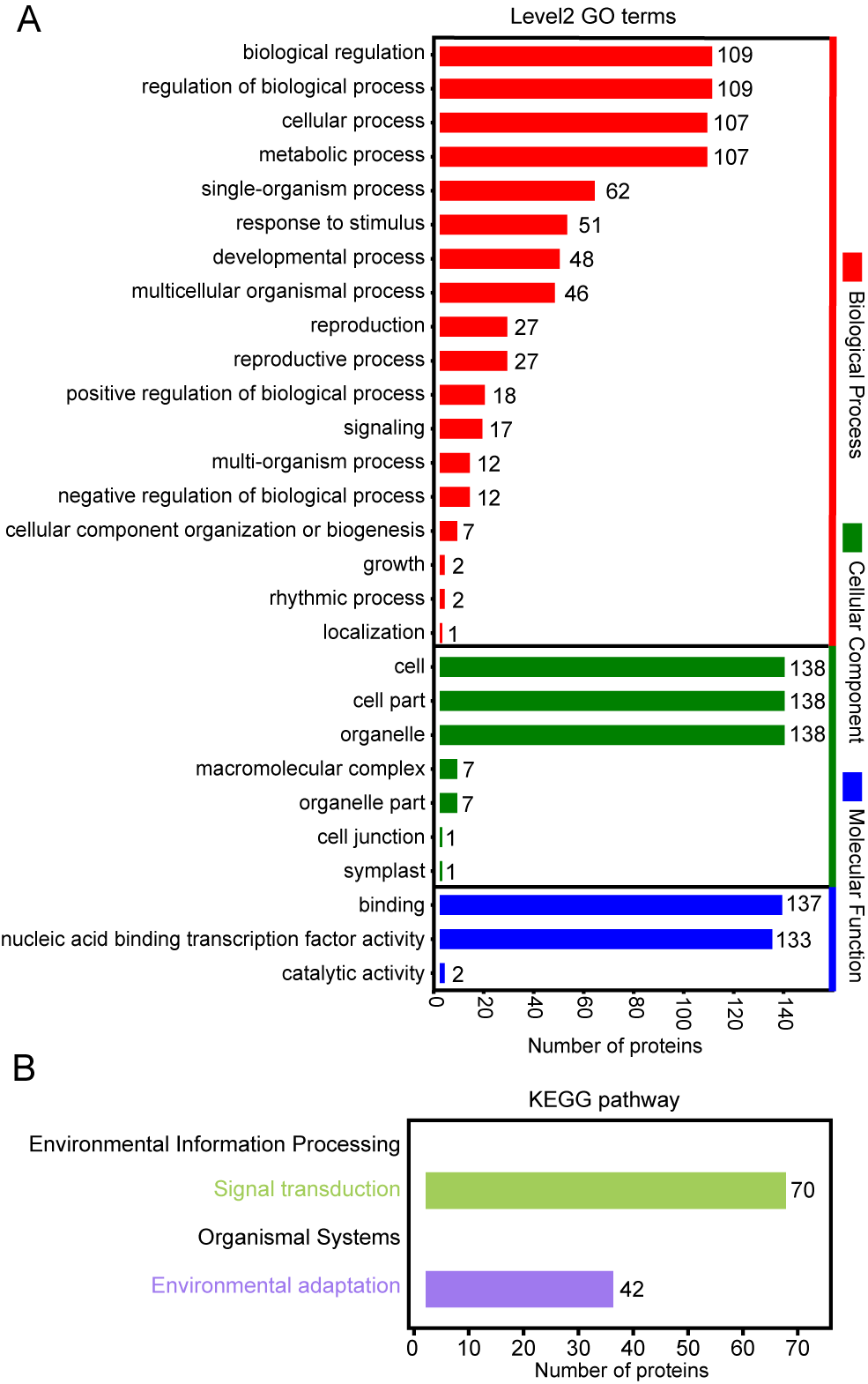


Figure S6 The functional enrichment analysis of GubHLH proteins. (A) Gene Ontology (GO) functional enrichment analysis of GubHLH proteins. (B) KEGG pathway enrichment analysis of GubHLH proteins.


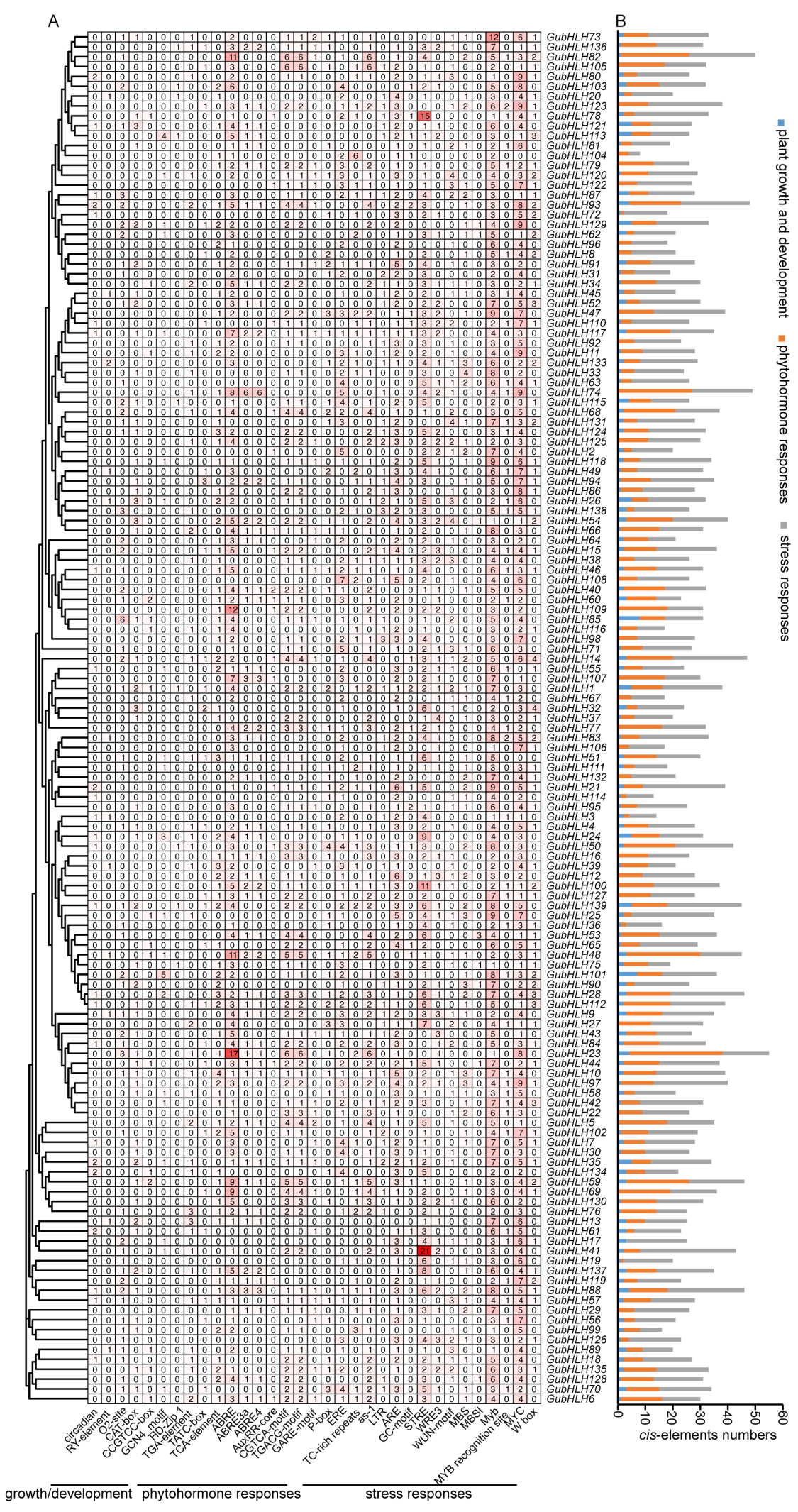


Figure S7 *Cis*-acting elements within the 2000 bp putative promoter region of the *GubHLHs*. (A) The heatmap of the numbers of *cis*-acting elements. The numbers in the grid showed the numbers of different *cis*-acting elements in the *GubHLHs*; (B) The different colored histogram represents the sum of the *cis*-acting elements of *GubHLHs* in each category.
