# Supplementary material for: Genome-wide identification and expression analysis of bHLH gene family revealed their potential roles in abiotic stress response, anthocyanin biosynthesis and trichome formation in Glycyrrhiza uralensis
Source: Front Plant Sci. 2025 Jan 21;15:1485757. doi: 10.3389/fpls.2024.1485757 (PMC11790457; doi:10.3389/fpls.2024.1485757)
Supplement: Supplementary file 6 [file Table5.docx]

| **Motif number** | **sequences** | **Function** |
| --- | --- | --- |
| Motif 1 | HSLAERRRRERJNERLKALRSLVPNGSKM | bHLH domain |
| Motif 2 | DKASMLDEAINYVKELQEQVK | bHLH domain |
| Motif 3 | CEKRPGLLLKLIQALESLRLD | unmatched |
| Motif 4 | EEPKSVYIHVRARRGQATDP | unmatched |
| Motif 5 | VTNSSVLPFGNSTLDITIIAQMDKEYSMTVKDLVKNLRQAL | unmatched |
| Motif 6 | SKSSLPEVEVRVSGKHVLJKI | unmatched |
| Motif 7 | EDVTDTEWFFLVSMSFSFPPGHGLPGKAYASRQHVWLSGA | bHLH-MYC and R2R3-MYB transcription factors N-terminal |
| Motif 8 | NTPAWSKWSNDGTEQQVAKLMEEDVGAAMQFLQSKALCIMPISLASAIYQ | unmatched |
| Motif 9 | LRPLVSSESWTYAIFWQL | bHLH-MYC and R2R3-MYB transcription factors N-terminal |
| Motif 10 | ILHLNVTTTDEIVLYSLSVKVEDGCKLGSVDEIAAAVHQLL | unmatched |

Table S5 Amino acid sequences and function of different motifs in GubHLH proteins
